# A dual L-glucose/L-galactose catabolic pathway in *Luteolibacter* species strain LG18

**DOI:** 10.1128/jb.00115-24

**Published:** 2025-10-16

**Authors:** Masashi Yachida, Yuki Shiratori, Shinya Iwabuchi, Tetsu Shimizu, Akira Nakamura

**Affiliations:** 1 Institute of Life and Environmental Sciences, University of Tsukuba 13121, Tsukuba, Ibaraki, Japan; 2 Microbiology Research Center for Sustainability (MiCS), University of Tsukuba 13121, Tsukuba, Ibaraki, Japan; 3 Tsukuba Institute for Advanced Research (TIAR), University of Tsukuba 13121, Tsukuba, Ibaraki, Japan; University of Massachusetts Chan Medical School, Worcester, Massachusetts, USA

**Keywords:** L-glucose catabolism, L-galactose catabolism, *Luteolibacter*, *Verrucomicrobiota*

## Abstract

The L-glucose catabolic pathway of *Luteolibacter* sp. strain LG18 was determined. L-glucose dehydrogenase (LguA) and L-gluconate dehydrogenase (LguD), purified from the cell extract of strain LG18, convert L-glucose to 5-keto-L-gluconate via L-gluconate, and these recombinant enzymes also utilize L-galactose and L-galactonate, respectively. Genes encoding these enzymes are both located in the gene cluster, *lguABCDEF,* which includes other genes possibly involved in L-galactose catabolism. After oxidation of L-gluconate, 5-keto-L-gluconate is converted to D-tagaturonate by LguG, a C-4 epimerase, determined with the recombinant enzyme. The subsequent LG18 reactions are likely to proceed in the same way as *Escherichia coli* L-galactonate catabolism, wherein LguC reduces C-5 to produce D-altronate that is dehydrated by LguB to produce 2-keto-3-deoxy-D-gluconate (KDG). LguH then phosphorylates KDG C-6 to produce KDG-6-phosphate, and an aldolase reaction driven by LguE produces D-glyceraldehyde-3-phosphate and pyruvate. Both *lguG* and *lguH* lie outside the *lguABCDEF* cluster, and LguH had a novel preference in utilizing pyrophosphate as a phosphate donor rather than ATP. Gene disruption studies indicated that, with the exception of *lguG*, which is involved only in L-glucose catabolism, the identified genes are indeed responsible for both L-glucose and L-galactose catabolism, indicative of a dual L-glucose/L-galactose catabolic pathway governed by a single set of genes. All the orthologs in this pathway are conserved in several *Luteolibacter* species, which also utilize L-glucose, suggesting that the same catabolic pathway is present in this genus.

**IMPORTANCE:**

L-glucose is presumably not present in natural environments, and to date, L-glucose catabolism has only been reported for a *Paracoccus laeviglucosivorans* strain 43P. The *Luteolibacter* strain LG18 differs taxonomically from 43P at the phylum level, and its L-glucose catabolic pathway differs from that of 43P at later steps from the C-4 epimerization reaction. In addition, most genes that drive LG18 L-glucose catabolism are also responsible for L-galactose catabolism, indicating the presence of a dual L-glucose/L-galactose catabolic pathway. This report contributes to a better understanding of homochirality in sugar catabolism, especially catabolism of glucose.

## INTRODUCTION

**Fig 1:**
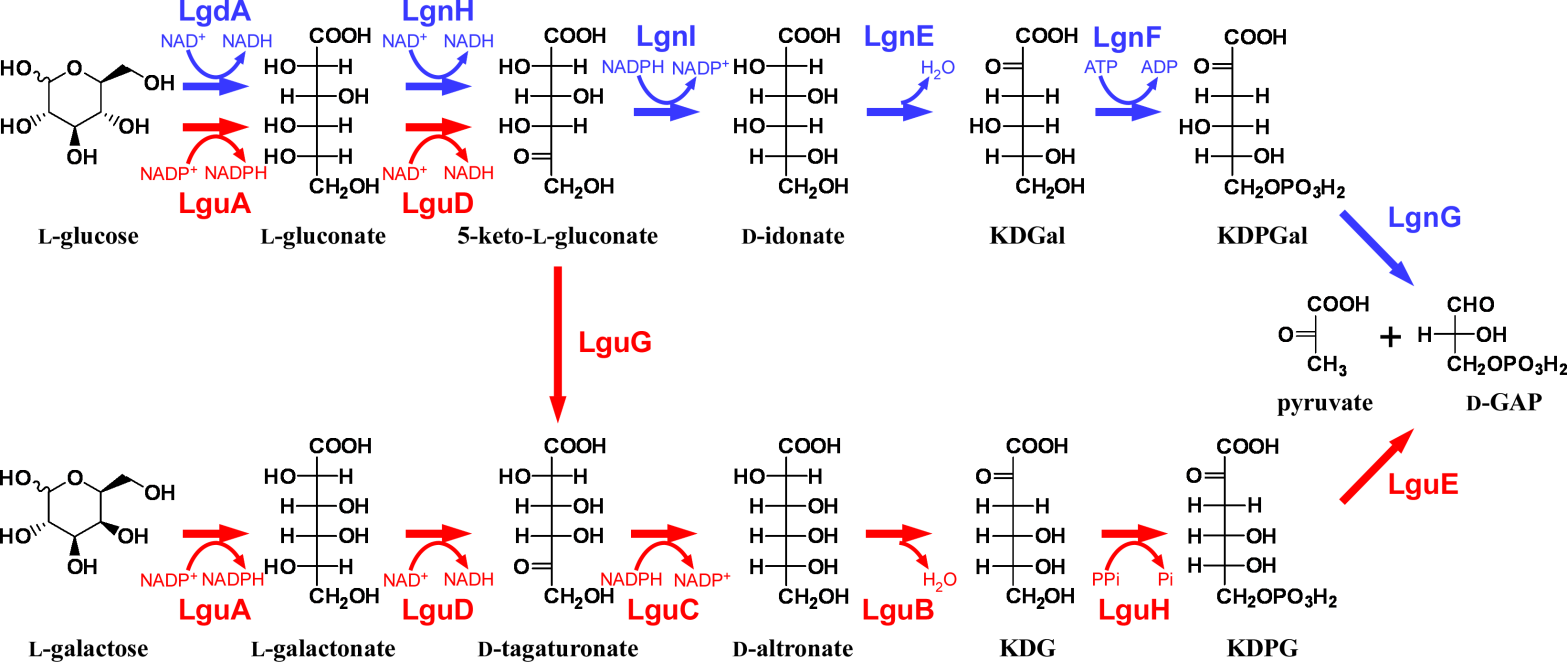
Model of the L-glucose/L-galactose catabolic pathway of LG18. The LG18 pathway and the related 43P pathway are shown in red and blue, respectively.

All living organisms show homochirality toward amino acids, with L-amino acids, but not D-amino acids, used for protein synthesis. A similar homochirality is present in sugars. D-glucose is the most abundant sugar in natural environments and can be metabolized by most organisms. In contrast, L-glucose is presumed not to be present in natural environments, and it had been thought that no organism can utilize L-glucose as an energy and carbon source (1); however, we previously isolated 43P, a *Paracoccus laeviglucosivorans* strain (2) belonging to the phylum *Pseudomonadota*, that can use L-glucose. Our characterization of the 43P L-glucose catabolic pathway (3; the 43P pathway) showed that L-glucose is converted to L-gluconate by L-glucose dehydrogenase (LgdA), followed by C-5 epimerization to D-idonate via 5-keto-L-gluconate mediated by L-gluconate dehydrogenase (LgnH)/5-keto-L-gluconate reductase (LgnI), to 2-keto-3-deoxy-D-galactonate (KDGal) by D-idonate dehydratase (LgnE), to KDGal-6-phosphate (KDPGal) by KDGal kinase (LgnF), and to D-glyceraldehyde-3-phosphate (D-GAP) and pyruvate by KDPGal aldolase (LgnG; Fig. 1). D-GAP and pyruvate can be used in later steps of glycolysis and the TCA cycle, respectively. The *lgdA* gene is part of a gene cluster putatively involved in inositol catabolism, whereas the *lgn* genes form an operon for L-gluconate catabolism (4). Since our report on 43P, to our knowledge, there have been no other reports of organisms that can use L-glucose.

We have isolated strain LG18 that can catabolize L-glucose and determined its complete genome sequence (5). Strain LG18 is classified into the genus *Luteolibacter*, belonging to the phylum *Verrucomicrobiota*. Therefore, strain LG18 differs taxonomically from strain 43P at the phylum level, suggesting that the L-glucose catabolic pathways of these two strains may differ.

Here, we analyzed the L-glucose catabolic pathway of strain LG18, termed the LG18 pathway. Although the LG18 and 43P pathways share the first two reactions that form 5-keto-L-gluconate, they diverge at the next reaction in which a C-4 epimerase in LG18 converts 5-keto-L-gluconate to D-tagaturonate, which is further converted to D-GAP and pyruvate in a similar manner as seen for *Escherichia coli* L-galactonate catabolism (6). We also demonstrate that most genes involved in the LG18 pathway are responsible for L-galactose catabolism as part of a dual L-glucose/L-galactose catabolic pathway carried out by enzymes encoded by a single set of genes.

## MATERIALS AND METHODS

### Strains, plasmids, and media

*Escherichia coli* DH10B was used for plasmid construction, and strain BL21 Star (DE3) (Invitrogen) and pET28-a(+) were used for recombinant protein production. pBBR1MCS-2 (7) and pUC19-mob (3) were used to construct conjugative plasmids for gene disruption, and *E. coli* S17-1 λ*pir* was used as a donor for biparental mating of cells. *E. coli* strains were cultured in LB medium supplemented with the indicated antibiotics.

Strain LG18 was cultured routinely in R2A medium, and a minimal medium (MM; per liter, 2.5 g carbon source, 0.54 g NH_4_Cl, 0.52 g KCl, 0.52 g MgSO_4_·7H_2_O, 1.06 g KH_2_PO_4_, 2.12 g K_2_HPO_4_, 2 mL Hutner’s trace element solution [per liter, 2.2 g ZnSO_4_·7H_2_O, 1.1 g H_3_BO_3_, 0.5 g MnCl_2_·4H_2_O, 0.5 g FeSO_4_·7H_2_O, 0.16 g CoCl_2_·6H_2_O, 0.16 g CuSO_4_·5H_2_O, and 0.11 g (NH_4_)_6_Mo_7_O_24_·4H_2_O], pH 6.8) with L-glucose (L-GlcMM), D-glucose (D-GlcMM), or L-galactose (L-GalMM).

*Luteolibacter ambystomatis* LMG 32214^T^, *L. arcticus* DSM 102244^T^, and *L. luteus* NBRC 114341^T^ were purchased from the respective culture collections.

### Measurements of cell growth and L-glucose consumption

Strains were pre-cultured in 5 mL D-GlcMM broth in ϕ18 mm test tubes (total volume, 27 mL) at 30°C for 2 days with shaking, and the cultures were inoculated into 10 mL L-GlcMM broth in ϕ25 mm test tubes (total volume, 63 mL) at 1% concentration. Culturing was conducted at 28°C with shaking, and cell growth was monitored by measuring the absorbance at 600 nm. The dinitrosalicylic acid method (8) was used to quantify the amount of L-glucose as reducing sugars in the culture supernatant.

### PCR amplification and sequencing

PCR primers used in this study are listed in Table S1, and amplification was conducted with Ex-Taq (Takara-Bio) and KOD FX Neo (Toyobo) polymerases. The cloned fragments were sequenced by dideoxy sequencing carried out by Eurofins Genomics.

### Enzyme assay substrates

L-glucose and L-galactose were purchased from Tokyo Chemical Industry. L-gluconate and D-galactonate were synthesized from L-glucose and D-galactose, respectively, by hypoiodite-in-methanol oxidation, as described previously (9). L-galactonate and D-idonate were prepared by base hydrolysis of L-galactono-1, 4-lactone and D-idono-1, 4-lactone (Biosynth), respectively.

5-Keto-L-gluconate and D-tagaturonate were synthesized as follows. A 10 mL reaction mixture containing 40 mM L-gluconate or L-galactonate, 2.5 mM NAD^+^, 100 mM ammonium carbonate, and 2.1 mg recombinant His_6_-LguD (production and purification of recombinant proteins are described below), as well as 55 mM 2-oxoglutarate, 0.5 mM ADP, and 2.7 mg of glutamate dehydrogenase from beef liver (Oriental Yeast Co., Ltd.) for regeneration of NAD^+^, was incubated for 3.5 h at room temperature. Then, 0.25 g activated charcoal was added to adsorb NADH and NAD^+^. The solution was further incubated at 37°C for 15 min and filtered with a 0.45 µm filter before passage over DOWEX 50W × 8 (H^+^ form, Fujifilm Wako) resin with 20 mM HCl. After neutralization of the solution with KOH, it was next applied to a DOWEX 1 × 8 (acetate form, Fujifilm Wako) column. The adsorbed reaction product was eluted with 100 mM potassium acetate and lyophilized. The lyophilizate was dissolved in 200 µL water before 1 mL ethanol was added to precipitate the product as a potassium salt. The precipitate was washed twice with ethanol and then three times with ethyl acetate, which was then evaporated.

KDGal was synthesized as previously described (3) with recombinant *E. coli* His_6_-DgoD. For KDPGal synthesis, a 20 mL reaction mixture containing 50 mM Tris-HCl, pH 7.5, 2 mM MgCl_2_, 40 mM KDGal, 80 mM ATP, and 1 mg recombinant His_6_-LgnF KDGal kinase (3) was incubated for 4 h at room temperature. Then, 5 g activated charcoal was added to adsorb ATP and ADP. The solution was filtered with a 0.22 µm filter and applied to a DOWEX 1 × 8 (chloride form) column that was washed with 80 mM HCl before the adsorbed KDPGal was eluted with 200 mM HCl. The solution containing KDPGal was neutralized with calcium carbonate, which was removed by filtration through a 0.22 µm filter, and lyophilized. The lyophilizate was dissolved in water, and ethanol was added to a final concentration of 90%. The precipitated KDPGal calcium salt was collected and washed three times with ethanol, which was evaporated.

Production of the above substrates was checked by thin-layer chromatography (TLC) or liquid chromatography-mass spectrometry (LC-MS), and purities were assessed as >90%. Other substrates were purchased from commercial sources, such as Sigma-Aldrich, Fujifilm Wako Chemicals, and Tokyo Chemical Industry.

### Detection of enzyme activities toward L-glucose

Enzyme activities toward L-glucose (kinases, isomerases, and dehydrogenases) in cell extracts of LG18 cultured in L-GlcMM broth for 48 h at 28°C were assayed as previously described (3). For kinase and isomerase activities, the cell extracts were incubated with 10 mM L-glucose in 100 mM Tris-HCl, pH 7.5, with or without 10 mM ATP and 1 mM MgCl_2_, at 25°C for 2 h. The reaction products were developed on a silica gel TLC plate (Merck) and detected with diphenylamine reagent (2 g diphenylamine, 2 mL aniline, and 20 mL 85% phosphoric acid in 100 mL acetone; 10). For dehydrogenase activity, the method described below was applied.

### Purification of L-glucose dehydrogenase (L-GDH) and L-gluconate dehydrogenase (L-GnDH)

LG18 was pre-cultured in 10 mL D-GlcMM broth in ϕ25 mm test tubes at 28°C for 48 h. The pre-culture was used to inoculate 1 L L-GlcMM broth in a 5 L culturing flask that was further cultured at 28°C for 48 h. Cells were harvested by centrifugation, washed, and resuspended in Buffer A (10 mM Tris-HCl, 1 mM DTT, 10% glycerol, pH 8.0). Cell-free extracts were prepared by sonication and centrifugation at 20,400 × *g* for 15 min at 4°C and used to purify L-GDH and L-GnDH. The procedures described below used prepacked columns with an ÄKTA pure 25 system (Cytiva). For TOYOPEARL DEAE chromatography, a BioLogic LP system (Bio-Rad) was used.

To purify L-GDH, cell-free extracts were applied to a TOYOPEARL DEAE-650M (Tosoh Bioscience; *ϕ* 2.5 cm × *d* 7 cm) column pre-equilibrated with Buffer A. After washing with Buffer A, the proteins were eluted with a linear gradient of 0 mM–500 mM NaCl in Buffer A. Fractions containing high L-GDH activity were pooled, and the same volume of Buffer B (50 mM Tris-HCl, 1 mM DTT, 10% glycerol, pH 8.0) containing 4 M (NH_4_)_2_SO_4_ was added. Next, the solution was applied to a HiTrap Butyl HP 5 mL column (Cytiva) pre-equilibrated with Buffer B containing 2 M (NH_4_)_2_SO_4_. After washing with the same buffer, proteins were eluted with a linear gradient of 2–0 M (NH_4_)_2_SO_4_ in Buffer B, and the active fractions were pooled and dialyzed against Buffer A containing 150 mM NaCl (Buffer C). Finally, the solution was applied to a HiTrap Q 1 mL column (Cytiva) pre-equilibrated with Buffer C. After washing with Buffer C, proteins were eluted with a linear gradient of 150 mM–450 mM NaCl in Buffer A, and the purified fractions were pooled.

To purify L-GnDH, cell-free extracts in Buffer D (10 mM Tris-HCl, 10% glycerol, pH 8.0) were used. First, anion-exchange chromatography was conducted with a TOYOPEARL DEAE-650M column as described above, using Buffer D as the basal buffer. Active fractions were pooled, and the same volume of Buffer E (50 mM sodium phosphate buffer, 10% glycerol, pH 7.0) containing 3 M (NH_4_)_2_SO_4_ was added to the solution. Hydrophobic chromatography was conducted with a HiTrap Butyl HP 5 mL column as described above, except using Buffer E as the basal buffer and eluting with a 1.5–0 M (NH_4_)_2_SO_4_ gradient. Next, the solution containing the active fractions was buffer-exchanged several times with Buffer D by ultrafiltration and dilution using an Amicon 10K (Merck) filtration device, and the solution was applied to a Resource Q 1 mL column (Cytiva) pre-equilibrated with Buffer D. After washing with Buffer D, the proteins were eluted with a linear gradient of 0 mM–500 mM NaCl in Buffer D. Fractions having high L-GnDH activity were pooled. Finally, the solution was buffer-exchanged and concentrated in Buffer F (20 mM sodium phosphate buffer, 10% glycerol, pH 7.0) by ultrafiltration and dilution using an Amicon 10K filtration device before application to a Superdex 200 10/300 GL column (Cytiva) pre-equilibrated with Buffer F. Proteins were eluted with Buffer F at a flow rate of 0.5 mL/min, and the purified fractions were pooled.

The purification steps were monitored by SDS-PAGE as described by Laemmli (11), and the protein amounts were determined with a BCA protein assay kit (Thermo Scientific), using bovine serum albumin as a standard.

### Determination of N-terminal amino acid sequences

Purified L-GDH and L-GnDH were resolved by 10% SDS-PAGE and electroblotted onto a polyvinylidene difluoride membrane. The bands corresponding to the enzymes were excised and used for Edman degradation and sequencing carried out by Hokkaido System Science.

### Recombinant enzyme production and purification

The open reading frames (ORFs) for LG18 *lguA-H*, as well as *uxaB* and *uxaC* from *E. coli* W3110, which encode D-altronate oxidoreductase and uronate isomerase, respectively, were PCR-amplified from genomic DNA with appropriate primers (Table S1). The amplified PCR fragments contained *Nde*I sites at the initiation codons and *Xho*I sites just downstream of the termination codons. After *Nde*I-*Xho*I digestion, the fragments were cloned into pET28a. The *lguG* and *lguH* ORFs were cloned into *Nde*I-*Xho*I-digested pET28a using the Gibson assembly method (12) with an NEBuilder HiFi DNA Assembly Kit (New England Biolabs). To express *lgnF* and *dgoD*, previously constructed plasmids were used (3). All constructs carried an N-terminal His_6_-tag present in the pET28a vector.

*E. coli* BL21 Star (DE3) transformed with the plasmids was cultured at 30°C in LB broth containing 50 µg/mL kanamycin. When the optical density at 600 nm reached 0.6, isopropyl-β-D-thiogalactopyranoside (final concentration 0.1 mM) was added. Culturing then continued for 3 h before harvesting by centrifugation and disruption by sonication in 20 mM sodium phosphate, pH 7.4, 0.5 M NaCl, and 40 mM imidazole. The cell-free extracts were loaded onto a HisTrap FF crude column (1 mL; Cytiva), and the His_6_-tagged proteins were eluted with the above buffer containing 500 mM imidazole.

### Enzyme assays and kinetic analyses

L-GDH and L-GnDH activities were assayed routinely by measurement of the reduction of NADP^+^ and NAD^+^, respectively, at 340 nm and 25°C. The reactions were conducted with 10 mM L-glucose and 1 mM NADP^+^ in 10 mM Tris-HCl, pH 7.5, and with 20 mM potassium L-gluconate and 1 mM NAD^+^ in 100 mM Tris-HCl, pH 7.5, respectively.

His_6_-LguA (L-GDH; 0.1 µg) was incubated with 0 mM–250 mM L-glucose or 0 mM–5 mM L-galactose and 1 mM NADP^+^, or 0 mM–1 mM NADP^+^ and 100 mM L-glucose, in 100 µL 10 mM Tris-HCl, pH 7.5, at 25°C. For His_6_-LguD (L-GnDH), 2.5 µg of the enzyme was incubated with 0 mM–50 mM L-gluconate or L-galactonate and 1 mM NAD^+^, or with 0 mM–10 mM NAD^+^ and 20 mM L-gluconate, in 100 µL 10 mM Tris-HCl, pH 7.5, at 25°C. Reduction of NAD(P)^+^ under both conditions was monitored by the absorbance at 340 nm, and quantification was conducted using a molecular absorption coefficient (ε = 6,200 M^−1^ cm^−1^).

To measure D-tagaturonate reductase activity, His_6_-LguC (15 ng) was incubated with 0 mM–15 mM D-tagaturonate and 0.25 mM NADPH in 100 µL 100 mM Tris-HCl, pH 7.5, and oxidation of NADPH was monitored as described above. Activity toward 5-keto-L-gluconate was assayed with 0 mM–110 mM 5-keto-L-gluconate and 0.3 µg His_6_-LguC. For D-altronate dehydrogenase activity, 0.3 µg His_6_-LguC was incubated with 0 mM–50 mM D-altronate and 1 mM NADP^+^ in 100 µL 100 mM Tris-HCl, pH 7.5, and reduction of NADP^+^ was monitored. Activity toward D-idonate was assayed with 0 mM–80 mM D-idonate and 6 µg His_6_-LguC in 100 µL 100 mM Tris-HCl, pH 7.5.

The following LguE and LguH reactions were conducted with coupling enzymes, the amounts of which were added not to become rate-limiting.

To measure 2-keto-3-deoxy-D-gluconate-6-phosphate (KDPG) aldolase activity of His_6_-LguE, pyruvate and D-GAP reaction products were quantified by coupling reactions with L-lactate dehydrogenase (LDH) and D-GAP dehydrogenase (GAPDH), as described by Wong and Yao (13), respectively. Kinetic analysis was conducted as follows. His_6_-LguE (0.1 µg) was incubated with 0 mM–2.5 mM KDPG, 0.2 mM NADH, and 1.5 units of LDH (from chicken heart; Oriental Yeast) in 100 µL 100 mM potassium phosphate, pH 7.0, at 25°C. The amount of pyruvate produced was quantified by monitoring the concomitant oxidation of NADH.

For 2-keto-3-deoxy-D-gluconate (KDG) kinase activity of His_6_-LguH, a coupling reaction with His_6_-LguE and GAPDH was conducted. A reaction mixture containing 1.5 µg His_6_-LguH, 0.5 µg His_6_-LguE, 0.75 µg GAPDH (Sigma-Aldrich), 5 mM KDG, 1 mM NAD^+^, 1 mM MgCl_2_, and 0 mM–20 mM pyrophosphate (PPi) or 0 mM–2 mM ATP in 100 µL 100 mM potassium phosphate, pH 7.0, was incubated at 25°C, and generation of NADH was monitored as described above. The parameters toward KDG were also assayed with 10 mM PPi and 0 mM–10 mM KDG.

The activities of His_6_-LguB (D-altronate dehydratase), His_6_-LguG (5-keto-L-gluconate 4-epimerase), and His_6_-LguF (fructokinase) were determined by detection of the reaction products as described below.

### Identification of enzyme reaction products

Enzyme reaction products were mainly identified by LC-MS and LC-MS/MS analyses using an LCMS-8040 apparatus (Shimadzu) and a COSMOSIL HILIC column (Nakarai Tesque; *ϕ* 4.6 mm × *d* 150 mm). The column oven was set to 40°C, and the flow rate was 0.8 mL/min. The reactions were conducted with an excess amount of the enzymes and prolonged reaction times.

His_6_-LguA (1 µg) was incubated with 10 mM L-glucose and 1 mM NADP^+^ in 100 µL 50 mM Tris-HCl, pH 7.5, at 25°C for 3 h before 80 µL acetonitrile and 15 µL 100 mM ammonium formate were added to a 5 µL aliquot of the reaction mixture. After mixing and centrifuging at 17,400 × *g* for 5 min, 1 µL of the supernatant was used for LC-MS analysis in a mobile phase of 80:20 acetonitrile:100 mM ammonium formate. Product ions were detected in the negative ion mode by monitoring *m*/*z* = 195. LC-MS/MS analysis was conducted for the precursor ions having *m*/*z* = 195 using a 20 eV collision energy.

His_6_-LguD (1 µg) was incubated with 10 mM L-gluconate and 1 mM NAD^+^ in 100 µL 50 mM Tris-HCl, pH 7.5, at 25°C for 2 h. Sample preparation and detection of the product ions were conducted as above, with detection of product ions at *m*/*z* = 193. LC-MS/MS analysis was conducted for the *m*/*z* = 193 precursor ions with a collision energy of 10 eV.

The chirality of LguA and LguD reaction products was also analyzed using an LC-2000 Plus high performance liquid chromatography (HPLC) system equipped with an OR-2090 Plus optical rotation detector (Jasco), with the same column as above and a mobile phase of 50:50 acetonitrile:10 mM ammonium formate.

To analyze His_6_-LguG reaction products, a coupling reaction with His_6_-LguD (forward reaction) or His_6_-UxaB (reverse reaction) was conducted. For the forward reaction, 5 mM L-gluconate, 1 mM NAD^+^, 5.1 µg His_6_-LguG, and 5.0 µg of His_6_-LguD were used, and for the reverse reaction, 5 mM D-altronate, 1 mM NAD^+^, 5.1 µg of His_6_-LguG, and 4.2 µg of His_6_-UxaB were used. Both reactions were carried out in 30 µL 50 mM Tris-HCl, pH 7.5, with incubation at 25°C for 1 h. Sample preparation and detection of the product ions were done as above, with detection at *m*/*z* = 193. Reactions using a mixture without His_6_-LguD or His_6_-UxaB were also conducted, and detection was at *m*/*z* = 193 and 195.

To analyze His_6_-LguC reaction products, a coupling reaction with His_6_-UxaC was conducted for the forward reaction. The reaction mixture had 30 mM D-galacturonate, 5 mM NADPH, 1.2 µg of His_6_-LguC, and 5.4 µg of His_6_-UxaC in 50 µL 200 mM Tris-HCl, pH 7.5, with incubation at 25°C for 2 h. For the reverse reaction, a mixture of 5 mM D-altronate, 5 mM NADP^+^, and 1.2 µg His_6_-LguC in 10 µL 100 mM Tris-HCl, pH 7.5, was incubated at 25°C for 2 h. Product ions were detected by monitoring at *m*/*z* = 195 and 193, respectively.

His_6_-LguB (0.8 µg) was incubated with 5 mM D-altronate, 20 mM DTT, and 0.5 mM FeSO_4_ in 10 µL 100 mM Tris-HCl, pH 7.5, at 25°C for 1 h. Then, 70 µL acetonitrile and 27 µL 10 mM ammonium formate were added to 3 µL of the reaction mixture. After centrifugation, 1 µL of the supernatant was used for LC-MS analysis as above, with a 70:30 mobile phase of acetonitrile:10 mM ammonium formate and detection at *m/z* = 177. LC-MS/MS analysis was conducted for the precursor ions with *m*/*z* = 177 and a collision energy of 10 eV.

His_6_-LguH (2.8 µg) was incubated with 10 mM KDG, 1 mM MgCl_2_, and 10 mM ATP or PPi in 20 µL 100 mM Tris-HCl, pH 7.5, at 25°C for 4.5 h. Then, 50 µL acetonitrile and 45 µL 100 mM ammonium formate were added to 5 µL of the reaction mixture. After centrifugation, 1 µL of the supernatant was used for LC-MS analysis, using a 50:50 mobile phase of acetonitrile:100 mM ammonium formate. Detection was conducted by monitoring *m/z* = 257.1 > 96.9 in multiple reaction monitoring (MRM) analysis.

The product of the His_6_-LguF reaction was detected by TLC. A reaction mixture with 1.6 µg His_6_-LguF, 10 mM fructose or related compounds, 5 mM ATP, and 0.5 mM MgCl_2_ was incubated in 20 µL 100 mM Tris-HCl, pH 7.5, at 25°C for 2 h. Then, 3 µL of the mixture was spotted onto a silica gel TLC plate (Merck) and developed in a mobile phase of a 6:1.5:1.5:1 mixture of ethyl acetate:methanol:acetate:water. After development, the plates were sprayed with the diphenylamine reagent, followed by heating at 120°C for 10 min. The spots were identified by their Rf values and colors with respect to authentic compounds.

### Gene disruption of strain LG18

Fragments including ~500 bp upstream and downstream of the target gene were PCR-amplified separately using respective up_fwd/up_rev and down_fwd/down_rev primer sets for each gene (Table S1). The kanamycin resistance (Km^r^) gene was obtained from pBBR1MCS-2 by PCR amplification using Pkan_F and kan_R primers (Table S1). An NEBuilder HiFi DNA Assembly Kit was used to clone the PCR fragments into *Hin*dIII-*Xba*I-digested pUC19-mob in the following order: upstream region, Km^r^ gene, and downstream region. The resulting plasmids were introduced into *E. coli* S17-1 λ*pir*.

LG18 was cultured in D-GlcMM broth at 28°C for 36 h, and *E. coli* S17-1 λ*pir* harboring a suicide plasmid for strain LG18 was cultured overnight at 37°C in LB broth containing 50 µg/mL kanamycin. Cells were washed twice, resuspended in sterile saline, and mixed at a 10:1 (donor:recipient) ratio before the mixture was dropped onto a polytetrafluoroethylene membrane placed on a D-GlcMM plate, which was incubated at 30°C for 8 days. Colonies were then collected and selected on D-GlcMM plates containing 50 µg/mL kanamycin and 10 µg/mL nalidixic acid at 30°C for 3 days. Gene disruption was confirmed by colony PCR using primer sets that recognized the corresponding flanking region (conF and conR for each gene; Table S1).

LG18 and its gene disruption mutants were used to assess L-glucose and L-galactose utilization. The strains were pre-cultured in 5 mL D-GlcMM broth in ϕ18 mm test tubes (total volume, 27 mL) at 30°C for 24 h with shaking, and the cultures were inoculated into L-GlcMM and L-GalMM broths at 1% concentration. Then, 150 µL of the broths were inoculated in a 300 µL 96-well microplate in triplicate and cultured at 30°C in a Synergy HTX microplate reader (BioTek) with shaking (567 cpm, 3 mm). Cell growth was monitored as described above at 2 h intervals. Doubling times of LG18 were calculated as previously described (14).

### Sequence similarity searches and phylogenetic analyses

The amino acid sequences of Lgu proteins were used for BLAST searches of the KEGG genome database, and the top 500 hits were considered. Lgu sequences were also used to identify protein families or relevant domains in the InterPro database, and the information was used to obtain the sequences of “reviewed” entries in the UniProt database. After selecting enzymes that act on sugars and sugar-related compounds from the UniProt entries, the obtained sequences were used for alignments using Clustal W in MEGA11 (15). Phylogenetic trees were compiled using the neighbor-joining method. After selecting one representative from a branch containing only those KEGG entries from the same genus, or a branch containing only the UniProt entries of the same enzyme function, phylogenetic analyses were conducted again, together with the sequences of the 43P pathway enzymes, and bvu:BVU_0219 and bvu:BVU_0222, which are reported to act as an L-galactose dehydrogenase and L-galactonate dehydrogenase (16), respectively.

## RESULTS

### Conversion of L-glucose to 5-keto-L-gluconate by LguA and LguD

**Fig 2:**
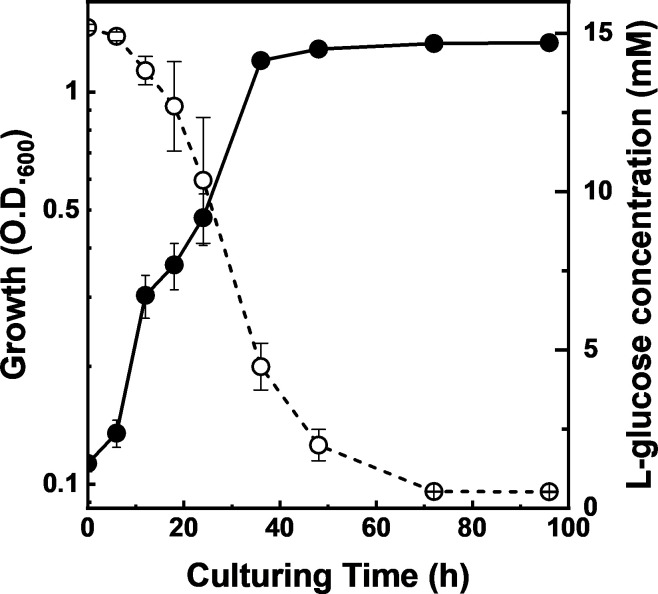
Growth and L-glucose consumption of strain LG18 in L-GlcMM. Growth and L-glucose consumption are indicated by closed and open circles, respectively. Average values ± SD for three independent cultures are shown.

**Fig 3:**
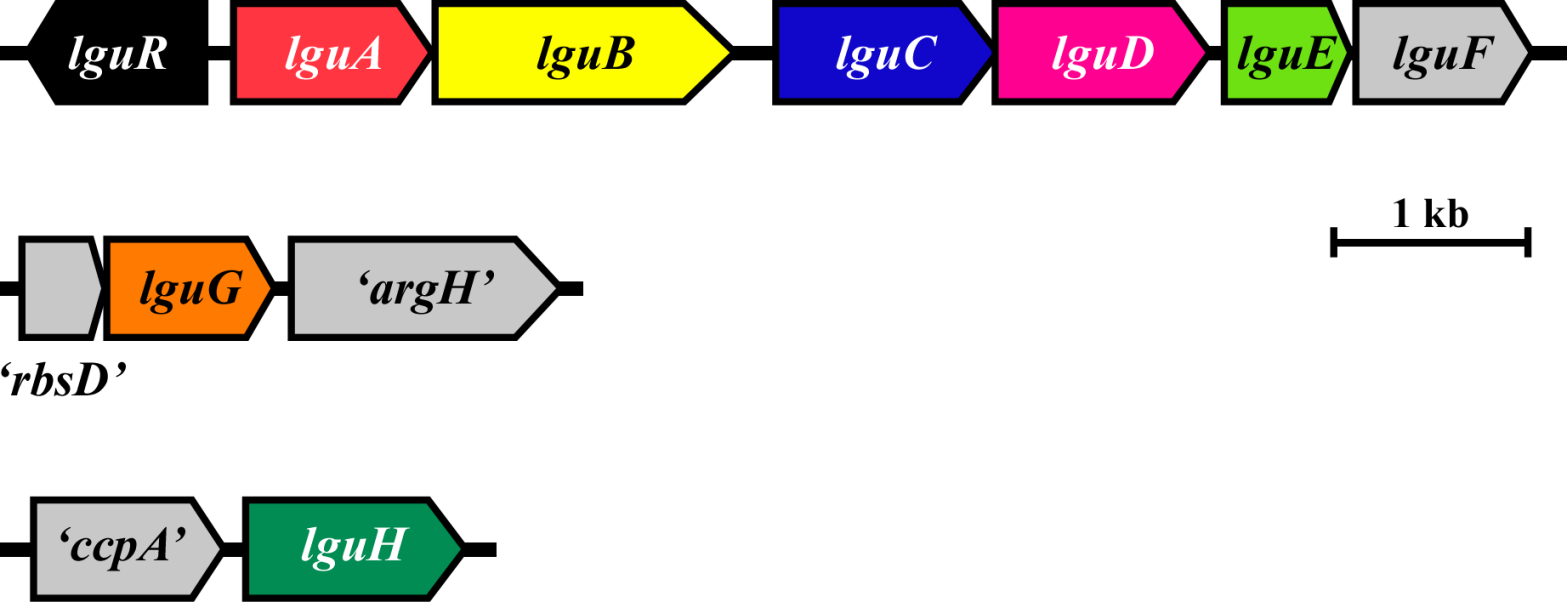
Gene organization of the *lgu* gene cluster (upper) and clusters containing *lguG* (middle) and *lguH* (lower). Genes encoding enzymes for L-glucose catabolism are colored, and other genes are shaded gray. The functions of the genes are *lguA*, NADP^+^-dependent L-glucose/L-galactose dehydrogenase; *lguB*, D-altronate dehydratase; *lguC*, NADPH-dependent D-tagaturonate reductase; *lguD*, NAD^+^-dependent L-gluconate/L-galactonate dehydrogenase; *lguE*, KDPG aldolase; *lguG*, 5-keto-L-gluconate 4-epimerase; and *lguH*, PPi-utilizing KDG kinase. Other genes’ functions annotated by the PROKKA program are *lguR*, HTH-type transcriptional activator Btr; *lguF*, fructokinase; “rbsD”, D-ribose pyranase; “argH”, argininosuccinate lyase I; and “ccpA”, catabolite control protein A. NCBI protein IDs are BCU78122, BCU78123, BCU78124, BCU78125, BCU78126, BCU78127, BCU78128, BCU76178, BCU76179, BCU76180, BCU78079, and BCU78078 for LguR, LguA, LguB, LguC, LguD, LguE, LguF, “rbsD,” LguG, “argH,” “ccpA,” and LguH, respectively.

Strain LG18 grew well in L-GlcMM broth with concomitant consumption of L-glucose (Fig. 2), indicating the presence of an L-glucose catabolic pathway. To identify the initial reaction toward L-glucose, crude extracts from LG18 cultured in L-GlcMM broth were used to detect activities of NAD(P)^+^-dependent dehydrogenases, kinases, and isomerases, which are known to be involved in sugar catabolism. Only an NADP^+^-dependent L-GDH activity was detected in the cell extracts, and the corresponding enzyme was partially purified (Table S2; Fig. S1A). The N-terminal amino acid sequence was determined to be METRKLGNTGLDVSV, and based on the genome sequence, the gene encoding L-GDH was identified as *llg_28380*, which was termed *lguA* (Fig. 3).

**Fig 4:**
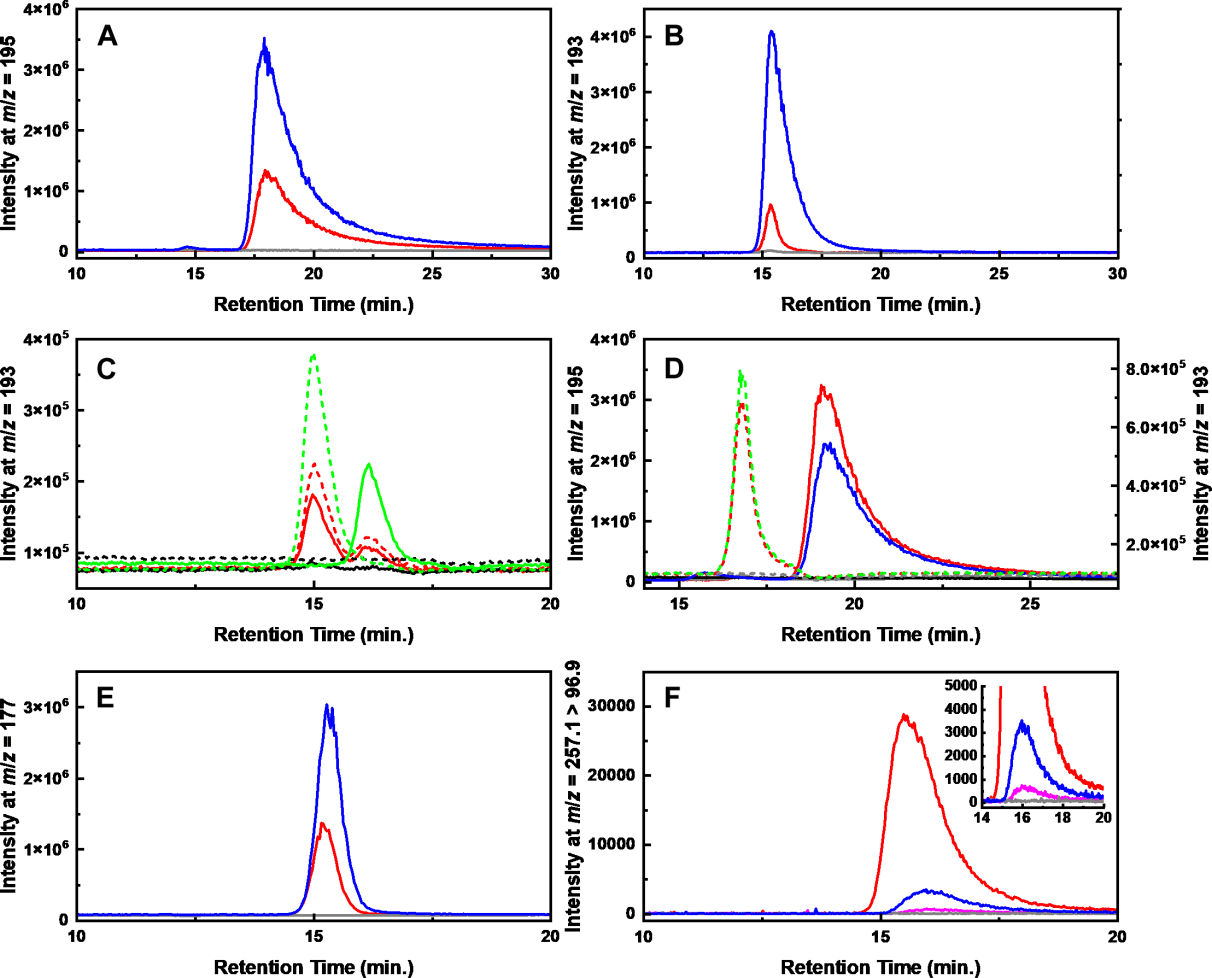
LC-MS analysis of His_6_-LguA (**A**), His_6_-LguD (**B**), His_6_-LguG (**C**), His_6_-LguC (**D**), His_6_-LguB (**E**), and His_6_-LguH (**F**) reaction products. Solid lines indicate chromatograms of the forward reaction products with (red or magenta) or without (gray, served as a negative control) corresponding enzymes, and blue lines indicate the authentic compounds L-gluconate (**A**), 5-keto-D-gluconate (**B**), D-altronate (**D**), KDG (**E**), and KDPG (**F**). The forward reactions were conducted with (**A**) L-glucose + His_6_-LguA, (**B**) L-gluconate + His_6_-LguD, (**C**) L-gluconate + His_6_-LguD + His_6_-LguG, (**D**) D-galacturonate + His_6_-UxaC + His_6_-LguC, (**E**) D-altronate + His_6_-LguB, and (**F**) KDG + His_6_-LguH + PPi (red) or ATP (magenta). Dashed lines in (**C**) and (D; right axis) indicate the reverse reaction products with (red) or without (gray, served as a negative control) corresponding enzymes. The reverse reactions were conducted with (**C**) D-altronate + His_6_-UxaB + His_6_-LguG and (**D**) D-altronate + His_6_-LguC. In (**C**), reactions of L-gluconate + His_6_-LguG (solid black) and D-altronate + His_6_-LguG (dashed black) were used as controls to show that these compounds are not directly produced from L-gluconate and D-altronate by His_6_-LguG, respectively. Solid and dashed green lines indicate the positions of substrates, D-tagaturonate and 5-keto-L-gluconate, generated by reactions of D-altronate + His_6_-UxaB and L-gluconate + His_6_-LguD, respectively, as well as no enzyme controls. In (**D**), the chromatogram of D-galacturonate + His_6_-LguC (solid black) was shown as another control to show that the product is not directly produced by His_6_-LguC. For the reverse reaction, the product of D-altronate + His_6_-UxaB was used to show the position of D-tagaturonate (dashed green). The inset in (**F**) shows an expanded view of the 14–20 min retention time during which faint production of KDPG with ATP was detected. Details of the analyses were described in Materials and Methods.

Using recombinant His_6_-LguA protein produced by an *E. coli* strain (Fig. S1C), enzymatic analyses were conducted. His_6_-LguA also showed activity toward L-galactose, a C-4 epimer of L-glucose. The kinetic analysis indicates that His_6_-LguA showed a 40-fold higher *k*_cat_/*K_m_* value for L-galactose over L-glucose, with a lower *K_m_* value toward L-galactose of about 80-fold (Table 1). No activity with D-idose, D-arabinose, D- and L-xylose, D-glucose, L-fucose, *myo*-inositol, and *scyllo*-inositol as substrates was observed, or for NAD^+^ as a coenzyme (<1.6 µmol min^−1^ mg protein^−1^). LC-MS analysis detected a peak at *m*/*z* = 195 for the LguA reaction product that corresponded to chemically synthesized L-gluconate based on its retention time. This peak was not produced in the absence of His_6_-LguA (Fig. 4A). The MS/MS spectrum for the precursor ion of *m*/*z* = 195 showed the same fragment ion spectrum as L-gluconate (Fig. S2A, a and b). Moreover, HPLC analysis with an optical rotation detector showed that the LguA product had identical optical rotation to L-gluconate (Fig. S2A, c). These results indicate that the product of the LguA-mediated reaction involving L-glucose is L-gluconate.

**TABLE 1:** Kinetic parameters for recombinant LG18 enzymes^*a*^

Enzyme	Substrate		K_m_	*k*_cat_	*k*_cat_/*K_m_*
	Fixed	Variable	(mM)	(s^−1^)	(s^−1^ mM^−1^)
LguA	NADP^+^	L-glucose	7.70 ± 0.49	54.6 ± 2.5	7.11
	NADP^+^	L-galactose	9.17 × 10^−2^ ± 0.93 × 10^−2^	24.9 ± 1.0	2.74 × 10^2^
	L-glucose	NADP^+^	7.93 × 10^−2^ ± 1.01 × 10^−2^	35.9 ± 1.7	4.57 × 10^2^
LguD	NAD^+^	L-gluconate	4.30 ± 0.34	2.17 ± 0.05	5.05 × 10^−1^
	NAD^+^	L-galactonate	2.96 ± 0.23	1.83 ± 0.03	6.18 × 10^−1^
	L-gluconate	NAD^+^	0.45 ± 0.03	2.63 ± 0.05	5.82
LguC	NADPH	D-tagaturonate	4.29 ± 0.70	535 ± 35	1.25 × 10^2^
	NADPH	5-Keto-L-gluconate	16.6 ± 2.1	102 ± 4	6.14
	NADP+	D-altronate	4.01 ± 0.41	8.97 ± 0.47	2.24
	NADP+	D-idonate	12.9 ± 2.8	1.05 × 10^−3^ ± 0.06 × 10^−3^	8.14 × 10^−5^
LguH	KDG	PPi	0.306 ± 0.063	2.93 ± 0.02	9.84
	KDG	ATP	0.055 ± 0.047	0.20 ± 0.06	5.61
	PPi	KDG	0.198 ± 0.012	3.49 ± 0.11	1.76 × 10^1^
LguE^*b*^		KDPG	0.137 ± 0.010	24.3 ± 0.5	1.77 × 10^2^

^*a*^ Assays were conducted in triplicate, and average values ± SD are shown.

^*b*^ Activity measured in terms of pyruvate production.

Next, an NAD^+^-dependent L-GnDH activity was detected in the crude extract of LG18 as an activity toward L-gluconate, and no activity was observed with NADP^+^ (< 1.6 × 10^−2^ μmol min^−1^ mg protein^−1^). The corresponding enzyme was purified (Fig. S1B; Table S3), and its N-terminal amino acid sequence was determined to be MKTLVLREPG. Its gene was identified as *llg_28410* in the genome sequence and was termed *lguD* (Fig. 3). The *lguD* gene is part of a gene cluster termed *lgu* that contains *lguA*. The other genes in the *lgu* cluster were annotated using the Prokka program (17) as D-altronate dehydratase (*lguB*), D-altronate oxidoreductase (*lguC*), KHG/KDPG aldolase (*lguE*), and fructokinase (*lguF*). Preceding the *lgu* gene cluster was a gene annotated to encode an HTH-type transcriptional activator, Btr, termed *lguR*, that had an opposite orientation.

Kinetic analyses using the purified His_6_-LguD protein (Fig. S1C) indicated that LguD utilized both L-gluconate and L-galactonate, with a 1.2-fold higher *k*_cat_/*K_m_* value for L-galactonate (Table 1). LC-MS/MS analysis of the LguD reaction product with L-gluconate showed the same retention time at *m/z* = 193 (Fig. 4B) and fragment ion spectrum as authentic 5-keto-D-gluconate (Fig. S2B, a and b). On HPLC, the product showed the opposite optical rotation relative to 5-keto-D-gluconate (Fig. S2B, c), indicating that the LguD reaction product is 5-keto-L-gluconate. Corresponding peaks were not detected for the reaction without His_6_-LguD.

Together, these results indicate that the first two reactions in the LG18 pathway are mediated by LguA and LguD to form 5-keto-L-gluconate via L-gluconate, which are the same as those in the 43P pathway.

### C-4 epimerization of 5-keto-L-gluconate by LguG

In the 43P pathway, downstream catabolism of 5-keto-L-gluconate proceeds by the reduction at the C-5 position to produce D-idonate. Based on gene annotation, LguC was initially assumed to act on 5-keto-L-gluconate to produce D-idonate. However, the catalytic efficiency of recombinant LguC toward 5-keto-L-gluconate was lower than that toward D-tagaturonate, the C-4 epimer of 5-keto-L-gluconate (see below). This result suggests that C-4 epimerization might occur as the next reaction in the LG18 pathway. A BLAST+ search of the LG18 genome using a query sequence from IolO, a 5-keto-L-gluconate 4-epimerase in *Thermotoga maritima* (18), identified three genes: *llg_08940*, *llg_23780*, and *llg_40790* annotated as D-tagatose 3-epimerase, which had amino acid sequence identities of 35, 30 and 28% to IolO, respectively. When recombinant *llg_08940* produced in *E. coli* was incubated with L-gluconate and His_6_-LguD, two peaks corresponding to 5-keto-L-gluconate and D-tagaturonate were detected at *m*/*z* = 193 in LC-MS analysis (Fig. 4C). Moreover, incubation of recombinant *llg_08940* with D-altronate and the D-altronate dehydrogenase His_6_-UxaB produced two peaks with the same retention times as 5-keto-L-gluconate and D-tagaturonate. In the absence of His_6_-LguD or His_6_-UxaB, no peaks were seen at *m*/*z* = 193; instead, only a peak corresponding to the substrate L-gluconate or D-altronate was detected at *m*/*z* = 195 (data not shown). Based on these results, we concluded that the product of *llg_08940*, which we term LguG (Fig. 3), is likely the third enzyme of the LG18 pathway and catalyzes a C-4 epimerization reaction that mediates interconversion of 5-keto-L-gluconate and D-tagaturonate. LguG did not react with L-gluconate and D-altronate, suggesting a preference for the 5-keto form.

### Pathway downstream of D-tagaturonate

As shown above, LguA, LguD, and LguG reactions can convert L-glucose to D-tagaturonate, which is also an intermediate compound in the L-galactose catabolic pathway of *Phocaeicola* (*Bacteroides*) *vulgatus* (16) and the L-galactonate catabolic pathway of *E. coli* (6). The annotations of most genes in the *lgu* gene cluster, except *lguF*, correspond to reactions in these pathways, suggesting that *lguB, lguC,* and *lguE* genes may be involved in a pathway downstream of D-tagaturonate. Enzyme assays were carried out with recombinant proteins to test this possibility.

#### LguC-mediated D-tagaturonate reduction

His_6_-LguC exhibited NADPH-dependent reductase activity toward D-tagaturonate and, in its reverse reaction, NADP^+^-dependent dehydrogenase activity toward D-altronate. In the reductase reaction, His_6_-LguC had a 20-fold higher *k*_cat_/*K_m_* value for D-tagaturonate over 5-keto-L-gluconate, whereas 2.8 × 10^4^-fold higher *k*_cat_/*K_m_* value for D-altronate over D-idonate was obseved in the dehydrogenase reaction (Table 1). His_6_-LguC had no activity towards L-galactonate and L-gluconate (<10 µmol min^−1^ mg protein^−1^; data not shown), the C-5 epimers of D-altronate and D-idonate, respectively, which reflects strict stereoselectivity for the D-form. Incubation of His_6_-LguC with D-galacturonate and His_6_-UxaC, the uronate isomerase for D-tagaturonate production, resulted in a peak at *m/z* = 195 on LC-MS that had the same retention time as authentic D-altronate (Fig. 4D). This peak did not appear in the absence of either His_6_-UxaC or His_6_-LguC. When His_6_-LguC was incubated with D-altronate, a peak having the same retention time as the His_6_-UxaB reaction product from D-altronate was seen at *m/z* = 193, and again this peak was detected only when His_6_-LguC was present. These results clearly demonstrated that LguC mediates interconversion between D-tagaturonate and D-altronate by C-5 reduction/dehydrogenation, and, as judged from the *k*_cat_/*K_m_* values, LguC prefers the reductase reaction. Therefore, we concluded that LguC functions as the fourth enzyme in the LG18 pathway to produce D-altronate from D-tagaturonate.

#### LguB-mediated dehydration of D-altronate

A TLC analysis showed that when His_6_-LguB was incubated with D-altronate, a spot with the same Rf value as authentic KDG could be detected, but no new spots were detected with D-idonate (data not shown). LC-MS/MS analysis revealed that the reaction product with D-altronate had the same retention time at *m/z* = 177 (Fig. 4E) and the same fragment ion spectrum as KDG (Fig. S2C). A corresponding peak was not detected in a reaction without His_6_-LguB. These results indicate that LguB converts D-altronate to KDG and functions as the fifth enzyme in the LG18 pathway.

#### LguH, not LguF, functions as a KDG kinase

Next, the function of the putative kinase LguF was observed. When His_6_-LguF was incubated with D-fructose and ATP, a new spot with the same Rf value and the same color as authentic D-fructose-6-phosphate was detected in a TLC analysis, whereas no reaction products were observed with KDG or KDGal (Fig. S2D). These results indicate that LguF is a fructokinase that is possibly not related to the LG18 pathway.

We found *llg_27930* that is located outside of the *lgu* gene cluster and was the sole gene annotated as a KDG kinase in the LG18 genome (Fig. 3). The reaction product of the recombinant protein, termed LguH, with KDG and ATP corresponded to a faint spot in a TLC analysis; the Rf value and color matched that of KDPG (data not shown), suggesting that LguH indeed functions as a KDG kinase. His_6_-LguH did not react with KDGal, showing substrate specificity toward KDG. We speculated that the weak activity was due to the choice of phosphate donor and subsequently conducted the reaction with PPi or ADP. An obvious spot corresponding to KDPG was observed in a TLC analysis with PPi, but not with ADP. Kinetic analysis with His_6_-LguE (see below) and a commercial GAPDH showed that the *K_m_* value for ATP was about 5.6-fold lower than that for PPi. In contrast, the *k*_cat_ value for PPi was about 14-fold higher than that for ATP, resulting in about 1.8-fold higher *k*_cat_/*K_m_* value with PPi (Table 1). Therefore, we concluded that, at least at the higher concentration, LguH preferentially utilizes PPi as a phosphate donor. Reaction product analysis by LC-MS revealed that the product had the same retention time as authentic KDPG at *m/z* = 257.1 > 96.9 (Fig. 4F). The peak height of the product obtained with PPi was about 30-fold higher than that with ATP, again suggesting a preference for PPi under our assay conditions. These results indicate that LguH is a PPi-utilizing KDG kinase and functions as the sixth enzyme in the LG18 pathway.

#### LguE-mediated aldol cleavage

Last, the function of LguE, annotated as a KHG/KDPG aldolase, was analyzed. A coupling reaction of His_6_-LguE with commercially available LDH showed consumption of NADH when KDPG was the substrate. This result is consistent with pyruvate production, and that with GAPDH showed production of NADH and, in turn, production of D-GAP. No activity was observed with KDPGal, the LgnG substrate in the 43P pathway (<1.6 µmol min^−1^ mg protein^−1^). The *k*_cat_/*K_m_* value of His_6_-LguE toward KDPG, measured as a coupling reaction with LDH, was about twofold higher than *E. coli* KDPG aldolase, Eda (19; Table 1). Therefore, we concluded that LguE functions as the seventh enzyme in the LG18 pathway to produce pyruvate and D-GAP.

### Involvement of the identified genes in the LG18 pathway

Finally, we conducted gene disruption studies to confirm that the identified genes are involved in L-glucose catabolism. We constructed disruption mutants of the *lgu* genes and paralogs of *lguG* (*llg_23780* and *llg_40790*), *lguC* (*llg_31090*), *lguB* (*llg_31080*), and *lguH* (*llg_41580* and *llg_28420*). Notably, when we initially attempted to obtain mutants using *E. coli* donor strains with shorter culturing time (<5 days) on D-GlcMM plates, most transconjugants selected via Km^r^ phenotype showed two bands corresponding to wild-type and disrupted genes in PCR analysis. These bands persisted after four rounds of single colony isolation on Km-supplemented D-GlcMM plates (data not shown). This result suggests that strain LG18 is a polyploid organism. However, culturing with the *E. coli* donor strains for 8 days did produce all the disruption mutants, except for the *lguE* paralog *llg_31200* (Fig. S3).

**Fig 5:**
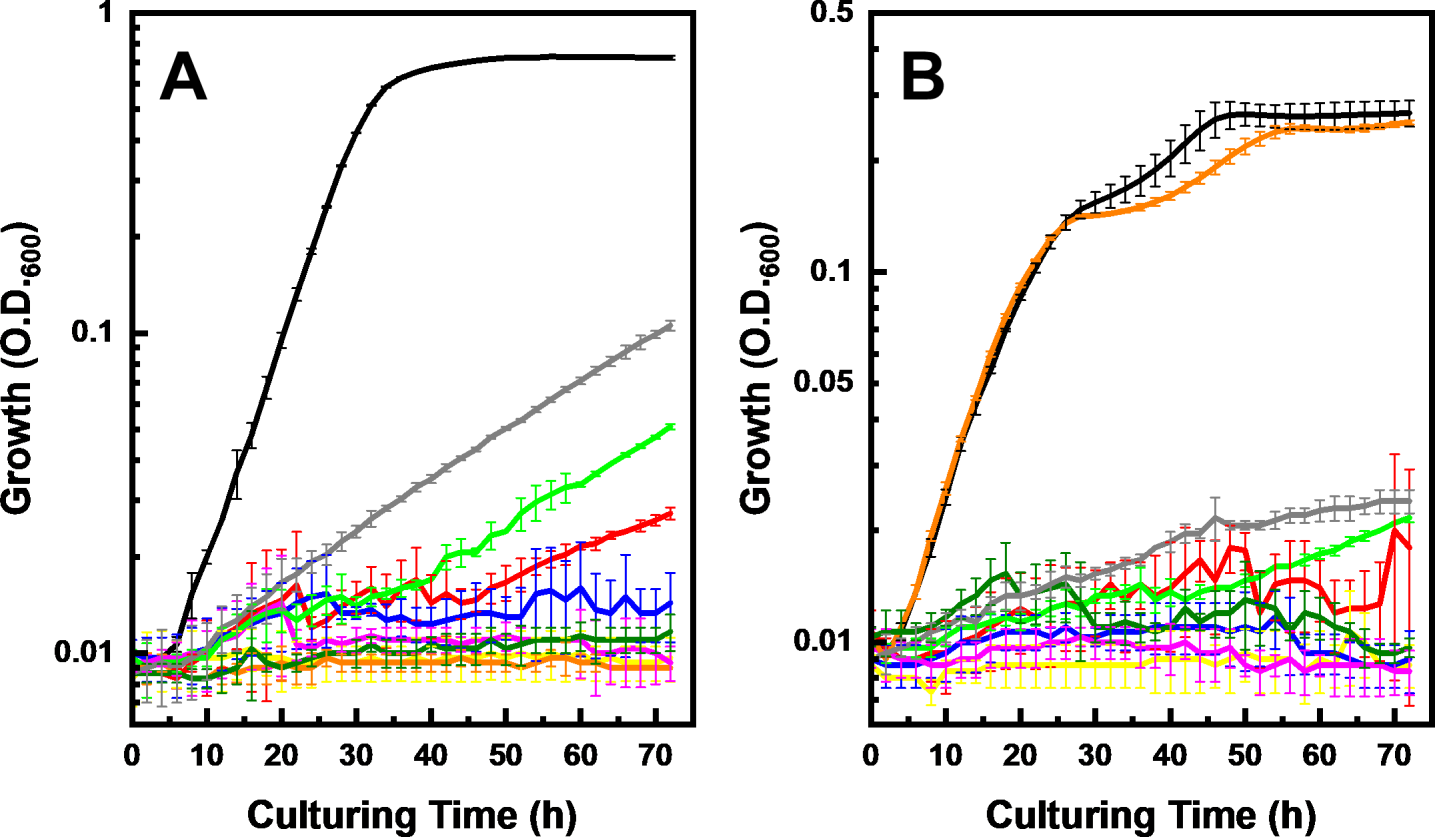
Growth of wild-type LG18 and its gene disruption mutants in L-GlcMM (**A**) and L-GalMM (**B**). Growth of wild-type LG18 (black), Δ*lguR* (gray), Δ*lguA* (red), Δ*lguD* (magenta), Δ*lguG* (orange), Δ*lguC* (blue), Δ*lguB* (yellow), Δ*lguH* (dark green), and Δ*lguE* (green) is shown. Values are the average ±SD of three independent cultures.

Interestingly, strain LG18 grew in L-GalMM as well as L-GlcMM (Fig. 5). Doubling times in L-GlcMM and L-GalMM were calculated to be 266 ± 5 and 306 ± 6 min, respectively, indicating a similar but slightly slower growth in L-GalMM. However, the maximum OD was different; in L-GlcMM, it reached >0.7, but only <0.3 in L-GalMM.

The *lgu* mutants, identified as above, showed growth defects in L-GlcMM, indicating that these genes are indeed involved in L-glucose catabolism (Fig. 5A). Moreover, the mutants, except for ∆*lguG*, showed growth defects in L-GalMM, indicating that these *lgu* genes, not *lguG*, also contribute to L-galactose catabolism (Fig. 5B). The growth defect observed for the ∆*lguR* in both the media suggested that LguR functions as a transcriptional activator for the *lgu* gene cluster. In addition, other mutants that had disruptions in paralogous genes, together with the ∆*lguF* mutant, did not show growth defects in either L-GlcMM or L-GalMM (Fig. S4). Because of a lack of a plasmid functional in this strain, we could not conduct a complementation assay by expressing the corresponding gene from a plasmid.

## DISCUSSION

This study sought to describe the L-glucose catabolic pathway in strain LG18, and a model for the LG18 pathway is shown in Fig. 1, together with the 43P pathway. The first two reactions are the same in the LG18 and 43P pathways, but the third reaction differs. In the 43P pathway, 5-keto-L-gluconate is converted to D-idonate by C-5 reduction, followed by dehydration, phosphorylation, and aldol cleavage. In contrast, the LG18 pathway involves C-4 epimerization and then proceeds in an analogous manner as the 43P pathway with corresponding substrates that instead have C-4 epimerization. Involvement of C-4 epimerization by LguG was supported by our gene disruption study (Fig. 5) and substrate specificity of LguC (Table 1). Gene disruptions in the 43P pathway were previously shown not to affect L-galactonate catabolism (3), suggesting that the *lgn* genes are responsible for L-gluconate catabolism only, and that catabolism proceeds using substrates having *4S* stereo configurations. On the other hand, LguC, LguB, LguH, and LguE in the LG18 pathway showed strong preferences for D-tagaturonate, D-altronate, KDG, and KDPG, respectively, indicating that catabolism downstream of the LguC reaction proceeds with substrates having *4R* stereo configurations. Therefore, the reactions downstream of D-tagaturonate and 5-keto-L-gluconate in the LG18 and 43P pathways, respectively, are analogous, yet distinct in terms of C-4 stereo configurations.

Since LguA and LguD can also utilize L-galactose and L-galactonate as substrates, respectively, the LG18 pathway described here can be recognized as a dual L-glucose/L-galactose catabolic pathway, as was confirmed by gene disruption studies (Fig. 5). The ∆*lguR*, ∆*lguE*, and ∆*lguA* mutants showed slow but distinct growth, especially in L-GlcMM. Consistent with the annotation, LguR seems to act as a transcriptional activator for the *lgu* gene cluster, and therefore slow growth of the ∆*lguR* mutant is explained by basal level of expression without LguR. Growth of the ∆*lguE* mutant can be explained by the presence of a paralog, *llg_31200*, which showed 98% identity and could not be disrupted. The reason for the slow growth in the ∆*lguA* mutant is unclear, because there are no genes showing high identities to *lguA* in the LG18 genome. For the complementation assays, we have tried to introduce pBBR1MCS-2 into strain LG18, either by transformation or transconjugation, but we could not obtain any colonies harboring the plasmid. As the *mob* and Km^r^ genes in this plasmid were functional in our gene disruption studies, this is possibly because the *ori* is not recognized by strain LG18.

Notably, though the doubling times of strain LG18 in L-GlcMM and L-GalMM were similar, the maximum OD in L-GlcMM was higher than that in L-GalMM. The precise reason for this discrepancy is not known; however, as LG18 utilizes both sugars with the same Lgu enzymes, it may be derived from the steps of incorporation into the cells.

The LG18 pathway appears to consist of L-galactose catabolic enzymes that do not have strict substrate specificities for the first two enzymes, LguA and LguD, and the LguG C-4 epimerase, a key enzyme for L-glucose utilization. In contrast, the first enzyme of the 43P pathway, LgdA, exhibits higher activity toward *scyllo*-inositol than L-glucose. The gene encoding LgdA is in a putative inositol-catabolic gene cluster, whereas genes encoding proteins that participate in later parts of the 43P pathway after L-gluconate are included in the *lgn* operon (4). Therefore, the 43P pathway can be assumed to consist of a *scyllo*-inositol dehydrogenase having loose substrate specificity and L-gluconate catabolic enzymes. The function of these pathways may be similar in terms of L-glucose catabolism, although the evolutionary origins of these pathways, as well as the substrate specificity of each enzyme, differ.

BLAST searches of the KEGG genome database using amino acid sequences of enzymes in the LG18 pathway indicated that, among organisms for which complete genome sequences are available, only two, *L. luteus* (luo) and *L. ambystomatis* (lamb), possessed all seven orthologs with high sequence identities. Additional searches of the NCBI database revealed that two other organisms, *L. arcticus* and *Luteolibacter rhizosphaerae,* also possessed these orthologs, indicating that only some species of the genus *Luteolibacter* appear to have an LG18-like pathway. Notably, we found no organisms containing six orthologs, such as those lacking *lguG* orthologs, that would putatively exhibit only L-galactose catabolism.

Phylogenetic analyses based on the amino acid sequences of Lgu enzymes and their related sequences (Fig. S5) showed that, in terms of high sequence identities, most orthologs in lamb and luo are very closely associated with the corresponding Lgu proteins, except for the LguD ortholog in luo that is located in a deeply branched nearby cluster. Analyses of orthologs from *L. arcticus* and *L. rhizosphaerae* indicated that these enzymes were closely associated with those of luo, including the positions of the LguD orthologs (data not shown). The phylogenetic positions of these orthologs supported the possibility that they may comprise the same L-glucose catabolic pathways as the LG18 pathway in these organisms. In fact, luo, lamb, and *L. arcticus* could grow in L-GlcMM and utilize L-glucose, but at a slower rate than that seen for LG18 (Fig. S6). In contrast, the enzymes conducting the corresponding steps in the 43P pathway are very distantly related, suggesting a different evolutionary origin than those in the LG18 pathways.

The clusters containing LguA, LguD, LguG, LguC, and LguH include sequences from the phyla *Bacteroidota* and/or *Planctomycetota*, whereas those from other phyla, such as *Bacillota* and *Actinomycetota* for LguA and LguH, respectively, are also included. Moreover, LguB is associated with a lineage containing sequences from the phylum *Pseudomonadota*, and LguE is associated with a lineage containing sequences from a broad range of phyla. Therefore, the evolutionary origins of Lgu enzymes appear to be divergent and less easily understood.

Comparison of the phylogenetic positions of Lgu enzymes with those of enzymes in the UniProt database for which functions are known indicated that most Lgu enzymes are distantly related to those having the same enzyme activity, such as L-galactonate dehydrogenase (bvu:BVU_0222), 5-keto-L-gluconate 4-epimerase (IOLO_THEMA), and enzymes in the *E. coli* L-galactonate catabolic pathway (LGOD_ECOLI, UXAB_ECOLI, UXAA_ECOLI, KDGK_ECOLI and ALKH_ECOLI). However, LguA is included in a cluster having the known L-galactose dehydrogenases, GALDH_ARATH and bvu:BVU_0219, suggesting that it evolved from a common ancestor. bvu:BVU_0219 and bvu:BVU_0222 can utilize, albeit very inefficiently, L-glucose and L-gluconate (16), respectively, although whether GALDH_ARATH can utilize L-glucose is unclear (20). Lineages with Lgu enzymes contain mainly the genome sequences listed in the KEGG genome database, and we could not determine the extent to which these enzymes have the same activities as the corresponding Lgu enzymes.

LguH is unique in its utilization of PPi as a phosphate donor. PPi-dependent kinases were reported for phosphofructokinase (21, 22), pyruvate phosphate dikinase (22), acetate kinase (23), *myo*-inositol 3-kinase (24), and phosphoenolpyruvate carboxykinase (25), but not for KDG kinases. Moreover, LguH is unique in its use of both ATP and PPi, in contrast to other enzymes that cannot use ATP. Whether LguH utilizes ATP under physiological conditions is unclear, particularly because the *K_m_* value toward ATP is about 5.6-fold lower than that for PPi, and the overall catalytic efficiencies (*k*_cat_/*K_m_*) are almost the same.

In conclusion, LG18 and 43P share the common physiological characteristic of L-glucose catabolism, but the catabolic pathways differ. Moreover, these strains also differ taxonomically at the phylum level. These findings lead us to presume that more (micro)organisms can utilize L-glucose, likely via other pathways than those described here.

## SUPPLEMENTAL MATERIAL

Supplemental figures and tables jb.00115-24-s0001.pdf Table S1 to S3 and Figures S1 to S6.

ASM does not own the copyrights to Supplemental Material that may be linked to, or accessed through, an article. The authors have granted ASM a non-exclusive, world-wide license to publish the Supplemental Material files. Please contact the corresponding author directly for reuse.
