# Supplementary material for: A dual L-glucose/L-galactose catabolic pathway in Luteolibacter species strain LG18
Source: J Bacteriol. 2025 Oct 16;207(11):e00115-24. doi: 10.1128/jb.00115-24 (PMC12632266; doi:10.1128/jb.00115-24)
Supplement: Supplemental figures and tables — Table S1 to S3 and Figures S1 to S6. [file jb.00115-24-s0001.pdf]

**Supplementary information**

# A dual L-glucose/L-galactose catabolic pathway in *Luteolibacter* species strain LG18

Masashi Yachida, Yuki Shiratori, Shinya Iwabuchi, Tetsu Shimizu, and Akira Nakamura

**Table S1. Primers used in this study.**

| Primer | Sequence (5'-3')[a] |
|---|---|
| **Primers used to construct expression plasmids** | |
| lguA_FNde | GCGC*CATA**TG***GAATATCGCAAATTGGGCAAC |
| lguA_RXho | GCGC*CTCGAG***TCA**AGCTCGGGCGGCCAG |
| lguB_FNde | GCGC*CATA**TG***AAACGTCTGGTCCAGGTCC |
| lguB_RXho | GCGC*CTCGAG***TCA**CAACGTCACACCCTGTTTCC |
| lguC_FNde | GCGC*CATA**TG***TCACTTCCTGAAACCATCCTTC |
| lguC_RXho | GCGC*CTCGAG***TCA**TGAGAGGATCGAGGAAAGG |
| lguD_Nde | GCGC*CATA**TG***AAGACCCTTGTACTCCGC |
| lguD_RXho | GCGC*CTCGAG***TCA**AAACCCGATCATCGCCTTG |
| lguE_FNde | GCGC*CATA**TG***ATCGACCGCATTCTCGCC |
| lguE_RXho | GCGC*CTCGAG***TCA**GACCTCCGCGGCGGC |
| lguF_Nde | GCGC*CATA**TG***AGCGAGTCGAAAGCATTGC |
| lguF_RXho | GCGC*CTCGAG***TCA**ATGGTTCGACGCCAGCAG |
| lguG_fwd | ctggtgccgcgcggcagc*catatg***ATG**AGCCACGCGCCATCC |
| lguG_rev | agtggtggtggtggtggtg*ctcgag***TCA**AACCGCCGCGAACGC |
| lguH_fwd | ctggtgccgcgcggcagc*cat**atg***CTCCAGATCAAGCCCAAG |
| lguH_rev | agtggtggtggtggtggtg*ctcgag*GAAATACCGGATGTCGGG |
| uxaB_NdeF | GGCCC*CATA**TG***AAAACACTAAATCGTCGCGA |
| uxaB_XhoR | GAGAA*CTCGAG***TTA**GCACAACGGACGTACAGCTT |
| uxaC_NdeF | GGCCC*CATA**TG***ACTCCGTTTATGACTGA |
| uxaC_XhoR | GGACC*CTCGAG***TTA**GTTCAGTTCAATGGCGA |
| T7 promoter | TAATACGACTCACTATAGGG |
| T7 terminator | ATGCTAGTTATTGCTCAGCGG |
| **Primers used to construct suicide plasmids** | |
| lguRup_fwd | ctatgaccatgattacgccaagcttTGAAATTGATTCCTCCCGCG |
| lguRup_rev | gatagcccagtagctgacatTGGCCGCTGAAATCGAGG |
| lguRdown_fwd | ggttcgaaatgaccgaccaaTGTTGGAAAGCTGCGGCATC |
| lguRdown_rev | gctcggtacccggggatcctctagaTTCCAGAAATACCACGGCG |
| lguAup_fwd | ctatgaccatgattacgccaaTGGAATATCGCAAATTGGGC |
| lguAup_rev | gatagcccagtagctgacatCAGCGAGGTGTCGTTCAAC |
| lguAdown_fwd | ggttcgaaatgaccgaccaaCCTACTTCAAGGAGAAGGG |

| | |
|---|---|
| lguAdown_rev | gctcggtacccggggatcctATCTCCTCAAGCACCAGC |
| lguBup_fwd | ctatgaccatgattacgccaagcttATGACAACGTGGCCGTCG |
| lguBup_rev | gatagcccagtagctgacatCAGCGTGTTGTTCTCGCAG |
| lguBdown_fwd | ggttcgaaatgaccgaccaaTTGGTTCAACGCCCAGGAG |
| lguBdown_rev | gctcggtacccggggatcctctagaATCTTCAGCGTCGGCACC |
| lguCup_fwd | ctatgaccatgattacgccaagcttAACCGCGAATGAACGCGAATG |
| lguCup_rev | gatagcccagtagctgacatTTGTTCTCGATCAGCTCGC |
| lguCdown_fwd | ggttcgaaatgaccgaccaaTGAACTGGATCACGGACG |
| lguCdown_rev | gctcggtacccggggatcctctagaACAAGGGTCTTCATGAGAG |
| lguDup_fwd | ctatgaccatgattacgccaaTGAAGACCCTTGTACTCCG |
| lguDup_rev | gatagcccagtagctgacatATGACCAGCGCGAACTCG |
| lguDdown_fwd | ggttcgaaatgaccgaccaaCGATCGTGCTCGATATCAATGAAG |
| lguDdown_rev | gctcggtacccggggatcctCGCACACGGTCCTACTGAAC |
| lguEup_fwd | ctatgaccatgattacgccaagcttATGATCCGAATTTCCACCGC |
| lguEup_rev | gatagcccagtagctgacatAGAACTGGAGGCCCACCTC |
| lguEdown_fwd | ggttcgaaatgaccgaccaaTTCCGAAGGCTGCAAGCTG |
| lguEdown_rev | gctcggtacccggggatcctctagaATGGTGATGTAAGCCGCG |
| lguFup_fwd | ctatgaccatgattacgccaagcttAAGGCTGCAAGCTGCTGAAG |
| lguFup_rev | gatagcccagtagctgacatACATCGGTATCGAGCGTG |
| lguFdown_fwd | ggttcgaaatgaccgaccaaACGCGGCTTACATCACCATC |
| lguFdown_rev | gctcggtacccggggatcctctagaACCGTGGAAACCCAATCC |
| lguGup_fwd | ctatgaccatgattacgccaagcttACATTTCCCTGACCCACG |
| lguGup_rev | gatagcccagtagctgacatGTGCTTCACCATGCCTGC |
| lguGdown_fwd | ggttcgaaatgaccgaccaaTTTCCCTGACCGATCCCG |
| lguGdown_fwd | gctcggtacccggggatcctctagaGTAATCGGGGATGGTGCG |
| lguHup_fwd | ctatgaccatgattacgccaagcttAACCCTACACGTGTAGGATTC |
| lguHup_rev | gatagcccagtagctgacatTACTTGTTGGCGGCCTTC |
| lguHdown_fwd | ggttcgaaatgaccgaccaaTCGTTTCCTACGACCTCAAC |
| lguHdown_rev | gctcggtacccggggatcctctagaTTGGAAATACCGGATGTCG |
| llg_23780up_fwd | ctatgaccatgattacgccaagcttGTCCTCCTTTGCAATCGAATAGAG |
| llg_23780up_rev | gatagcccagtagctgacatAAGCGGTTGATCGGCTCG |
| llg_23780down_fwd | ggttcgaaatgaccgaccaaACCGACCTCGTCAACAAC |
| llg_23780down_rev | gctcggtacccggggatcctctagaTTCGTAGCTGGTGTAGCG |

| | |
|---|---|
| llg_31080up_fwd | ctatgaccatgattacgccaagcttGAAGATCCATTGCCAAGC |
| llg_31080up_rev | gatagcccagtagctgacatACTCTTCAGCGTGTTGTTC |
| llg_31080down_fwd | ggttcgaaatgaccgaccaaGATCGTTTCAAAGGCGAAG |
| llg_31080down_rev | gctcggtacccggggatcctctagaACGAAGAGACCCATCACAAC |
| llg_31090up_fwd | ctatgaccatgattacgccaagcttTCAACTCCTTTCGCATGTC |
| llg_31090up_rev | gatagcccagtagctgacatTTCACCAGCTCCAGCAAG |
| llg_31090down_fwd | ggttcgaaatgaccgaccaaATGGATCACGGACGAGTG |
| llg_31090down_rev | gctcggtacccggggatcctctagaGCCTTCCTGCAGAAGATG |
| llg_31200up_fwd | ctatgaccatgattacgccaagcttTCCATCTGGAGGGTCATTC |
| llg_31200up_rev | gatagcccagtagctgacatAGGCTTTCAACATCGGCAC |
| llg_31200down_fwd | ggttcgaaatgaccgaccaaGAATCACCTCCGCCAATC |
| llg_31200down_rev | gctcggtacccggggatcctctagaTCCAGTGGTAGAGCGGATTC |
| llg_40790up_fwd | ctatgaccatgattacgccaagcttGGATCTGACCTGCTCTCC |
| llg_40790up_rev | gatagcccagtagctgacatGTTCCATTCCGGTGCCAG |
| llg_40790down_fwd | ggttcgaaatgaccgaccaaCGTTTCGTTCTTCCAGAAG |
| llg_40790down_rev | gctcggtacccggggatcctctagaTGAAACACTCGTGGACGG |
| llg_41580up_fwd | ctatgaccatgattacgccaagcttGGTGATGGCGAACTTGATC |
| llg_41580up_rev | gatagcccagtagctgacatTCATAACACCGGGCGAGAAC |
| llg_41580down_fwd | ggttcgaaatgaccgaccaaGAAATGGCTTTGTCGCTC |
| llg_41580down_rev | gctcggtacccggggatcctctagaATCTGATCGCGTCCTTCAC |
| Pkan_F | ATGTCAGCTACTGGGCTATCTGGA |
| kan_R | TTGGTCGGTCATTTCGAACC |
| **Primer used to confirm gene disruption** | |
| lguRconF | TTTCGTCGCCAGCAGATAACTCTC |
| lguRconR | TTCCTCGGTGGATGTGAACCTG |
| lguAconF | TGACGAGTGGGCGGAAATGC |
| lguAconR | GCCGATGGGAAAGCCATACTTG |
| lguBconF | AACATCCGGGACAACATCGCCTAC |
| lguBconR | TCGAGGATCACCTTCATTAGCTCAC |
| lguCconF | TGAAGGTGATCCTCGATGTGG |
| lguCconR | AGGTGCCGCAGTTCATGTAG |
| lguDconF | ACCTGAACTCCTTTCCTCGATCCTC |
| lguDconR | ATGACGGTGATGGTGGTGGAG |

| | |
|---|---|
| lguEconF | TGGAGATCACGAACGGTGAC |
| lguEconR | CGACCTTGAAATGACCGAACTCC |
| lguFconF | ATGATCGACCGCATTCTCGCCAAG |
| lguFconR | TGTCGAGCATCGAGGTTTCGTTC |
| lguGconF | ACACCAACACCCTCGTCATTTC |
| lguGconR | CAAACGCCAGTCATACGAGATC |
| lguHconF | ATCGCCATCGGCATCATCAACC |
| lguHconR | TACCGCCAGGAGTTCGTGTTCAAG |
| llg_23780conF | TCGAACAGAAGCCGGAATTGG |
| llg_23780conR | TTGAGGCCGGTTTCCTTCAC |
| llg_31080conF | TTTGGGATTAGGTATCGGGTGG |
| llg_31080conR | ATGTGGAACGTGGGAACGAAG |
| llg_31090conF | ACATACGAAGAACCCGCGAAC |
| llg_31090conR | AATGGCATCGGTATCGGGAGTC |
| llg_31200conF | TTCTCAAGGCTCCTTCCTCTG |
| llg_31200conR | ATTCGCCTGATCCCAGATGC |
| llg_40790conF | CAAATACAAGGACTTCACGCTCG |
| llg_40790conR | TTCAAGACCTTCTGGGTGTCC |
| llg_41580conF | TGAATGGCCTGTGCTTCTGC |
| llg_41580conR | GTTCTACACCACGTCCGAAATGC |

[a]Restriction sites that were introduced are shown in italics; initiation and termination codons of each ORF are in bold. Lowercase texts indicate homologous arms to the vectors pET28a and pUC19-mob, and the $Km^r$ cassette for Gibson assembly.

**Table S2. L-Glucose dehydrogenase purification.**

| Purification step | Total protein (mg) | Total activity (μmol $min^{-1}$) | Specific activity (μmol $min^{-1}$ $mg^{-1}$) | Yield (%) |
|---|---|---|---|---|
| Cell-free extract | 98.3 | 18.7 | $1.90 \times 10^{-1}$ | 100 |
| TOYPEARL DEAE 650M | 25.7 | 14.8 | $5.75 \times 10^{-1}$ | 79.1 |
| HiTrap Butyl | $9.00 \times 10^{-1}$ | 12.1 | 13.4 | 64.7 |
| HiTrap Q | $9.1 \times 10^{-2}$ | 2.0 | 22.0 | 10.7 |

**Table S3. L-Gluconate dehydrogenase purification.**

| Purification step | Total protein (mg) | Total activity (μmol $min^{-1}$) | Specific activity (μmol $min^{-1}$ $mg^{-1}$) | Yield (%) |
|---|---|---|---|---|
| Cell-free extract | 126 | 2.45 | $1.94 \times 10^{-2}$ | 100 |
| TOYOPEARL DEAE 650M | 30.3 | 1.94 | $6.40 \times 10^{-2}$ | 79.2 |
| HiTrap Butyl | 2.77 | 1.11 | $4.01 \times 10^{-1}$ | 45.3 |
| Resource Q | $7.4 \times 10^{-2}$ | $3.0 \times 10^{-1}$ | 4.1 | 12.2 |
| Superdex 200 | $1.2 \times 10^{-2}$ | $6.2 \times 10^{-2}$ | 5.2 | 2.53 |

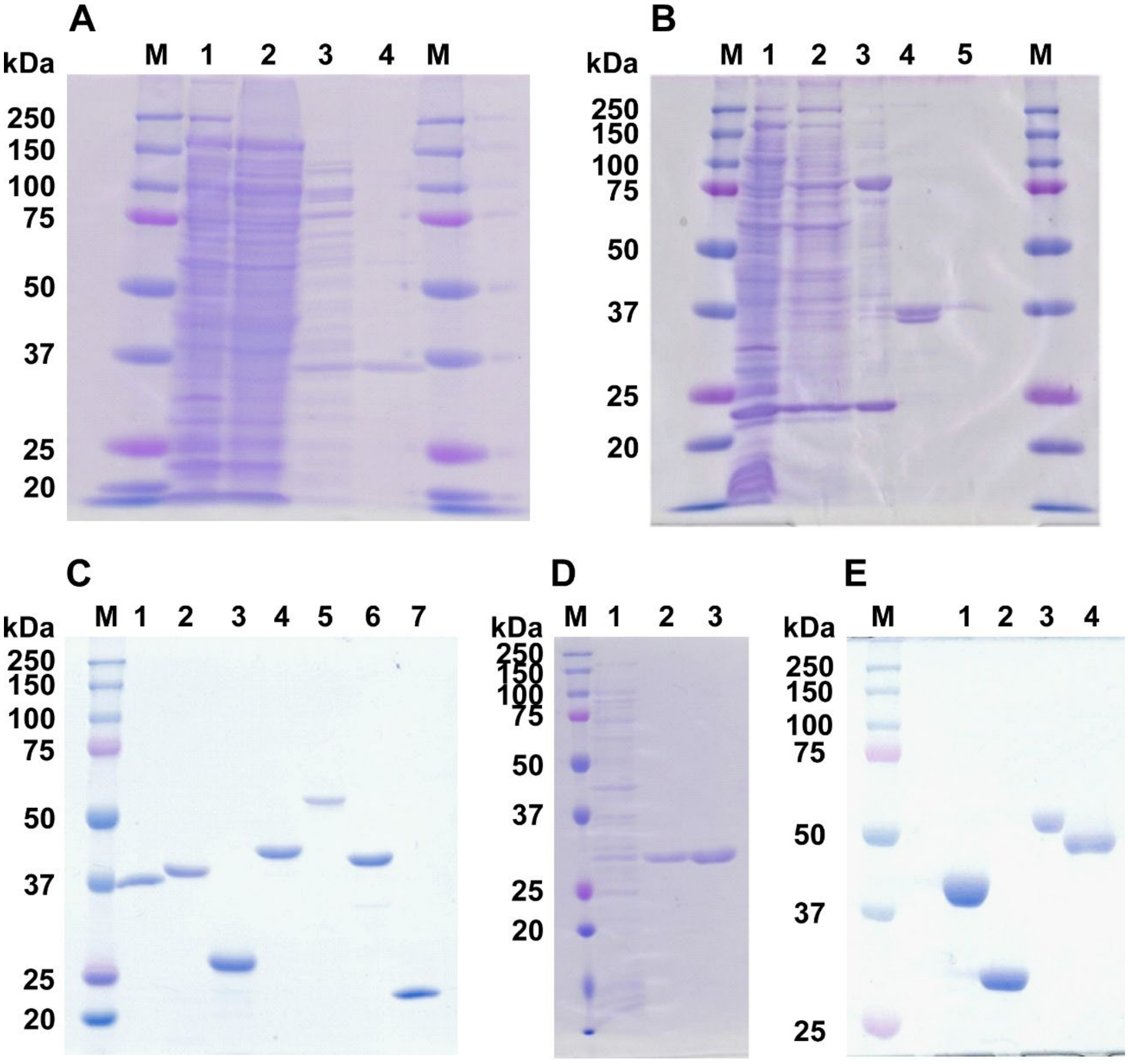


**Fig. S1. SDS-PAGE of purification steps of native L-glucose dehydrogenase (A) and native L-gluconate dehydrogenase (B) and purified recombinant enzymes (C-E).** Lane M shows molecular markers. (**A**) Lane 1, cell-free extracts; 2, after TOYOPEARL DEAE; 3, after HiTrap Butyl; and 4, after HiTrap Q chromatography. (**B**) Lane 1, cell-free extracts; 2, after TOYOPEARL DEAE; 3, after HiTrap Butyl; 4, after Resource Q; and 5, after Superdex 200 chromatography. (**C**) Lane 1, His$_6$-LguA; 2, His$_6$-LguD; 3, His$_6$-LguG; 4, His$_6$-LguC; 5, His$_6$-LguB; 6, His$_6$-LguH; and 7, His$_6$-LguE. (**D**) Lane 1, cell-free extracts of an *E. coli* strain expressing *His$_6$-lguF*; 2 and 3, purified His$_6$-LguF. (**E**) Lane 1, His$_6$-DgoD; 2, His$_6$-LgnF; 3, His$_6$-UxaB; and 4, His$_6$-UxaC.

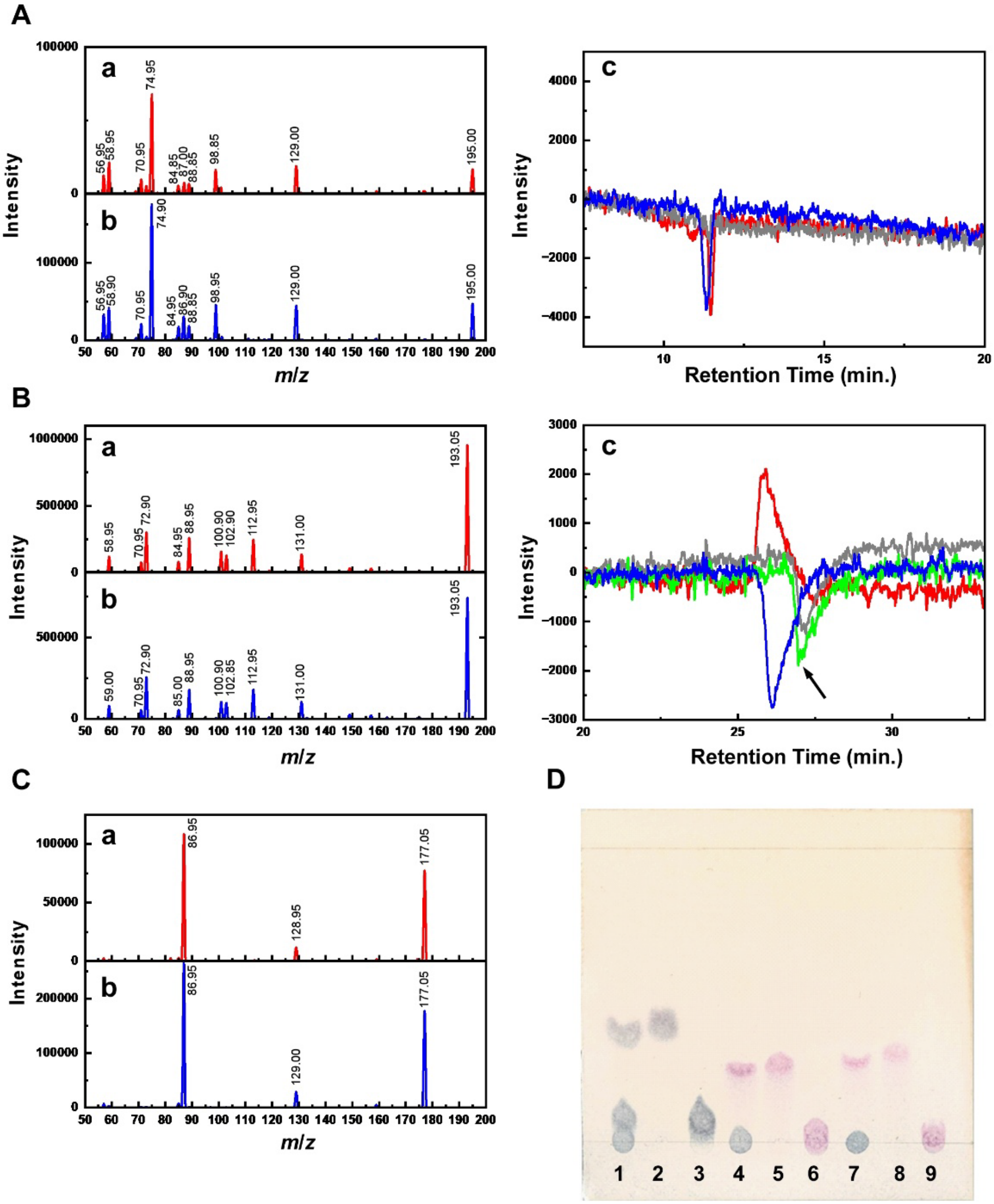


**Fig. S2. Identification of His$_6$-LguA (A), His$_6$-LguD (B), His$_6$-LguB (C), and His$_6$-LguF (D) reaction products.** (**A-C**) LC-MS/MS analysis of enzyme reaction products of corresponding enzymes are shown in (**a**), and those for authentic compounds L-gluconate (**A**), 5-keto-D-gluconate (**B**), and KDG (**C**) are shown in (**b**). Panel (**c**) shows HPLC detection of reaction products with a chiral detector. Red and gray lines indicate reactions with and without corresponding enzymes, respectively; blue lines correspond to authentic compounds L-gluconate (**A**) and 5-keto-D-gluconate (**B**). Green line in (**B**) indicates results for the L-gluconate substrate with the peak indicated by an arrow. (**D**) TLC analysis. Samples of His$_6$-LguF reactions with D-fructose, KDG, and KDGal were spotted onto lanes 1, 4, and 7, respectively, and D-fructose, D-fructose-6-phosphate, KDG, KDPG, KDGal, and KDPGal standards were spotted onto lanes 2, 3, 5, 6, 8, and 9, respectively. Green spots observed at the origin of lanes 1, 4, and 7 are ATP or ADP.

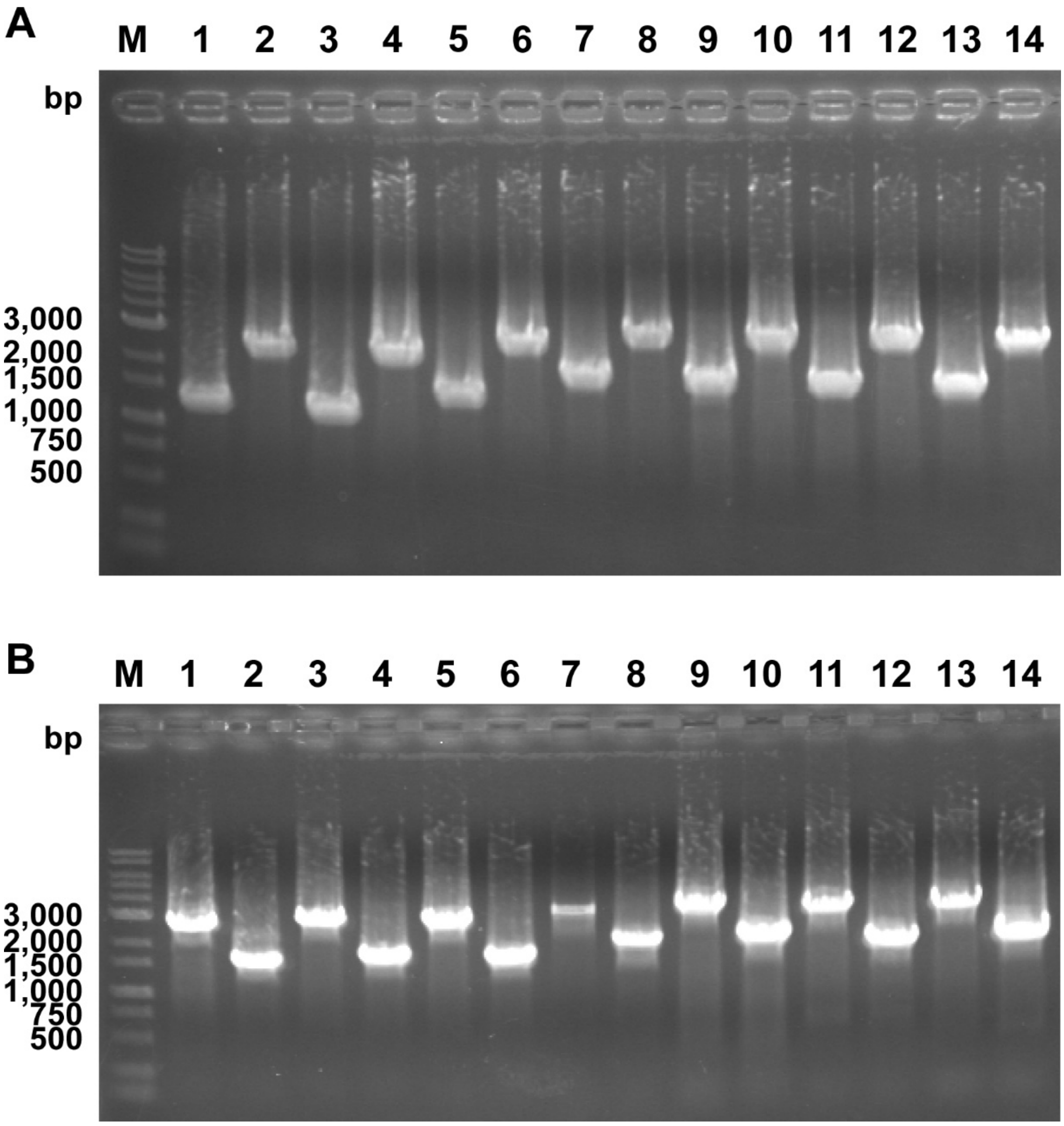


**Fig. S3. PCR analysis of gene disruption mutants.** (**A**) PCR reactions to amplify *lguA* (lanes 1 and 2), *lguD* (3 and 4), *lguG* (5 and 6), *lguC* (7 and 8), *lguB* (9 and 10), *lguH* (11 and 12), and *lguE* (13 and 14) were conducted with LG18 chromosomal DNA (odd numbers) and corresponding deletion mutants (even numbers). (**B**) PCR reactions to amplify *llg_70790* (lanes 1 and 2), *llg_23780* (3 and 4), *llg_31090* (5 and 6), *llg_31080* (7 and 8), *lguF* (9 and 10), *llg_41580* (11 and 12), and *lguR* (13 and 14) were conducted with LG18 chromosomal DNA (even numbers) and corresponding deletion mutants (odd numbers). Primers used in this study are shown in Table S1. Lanes M denote molecular mass markers.

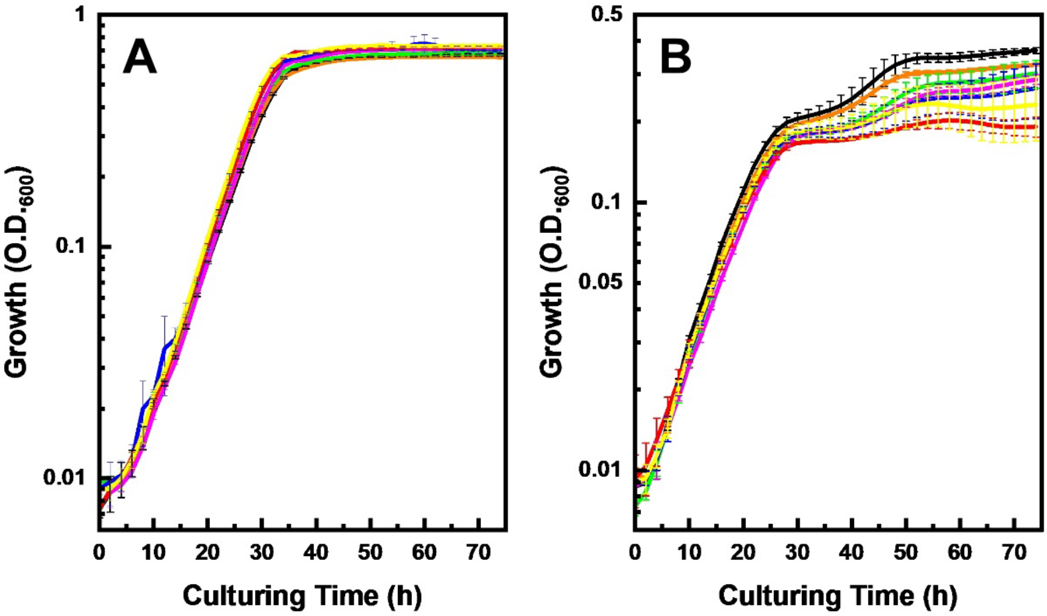


**Fig. S4. Growth of LG18 and gene disruption mutants paralogous to *lgu* genes in L-GlcMM and L-GalMM.** Growth of wild-type and gene-disrupted LG18 in L-GlcMM **(A)** and L-GalMM **(B)** is shown. Black, LG18; orange, *ΔlguF*; red and green, mutants deficient in paralogous genes to *lguG*, *llg_40790* and *llg_ 23780*, respectively; blue, mutant deficient in paralogous gene to *lguC*, *llg_31090*; magenta, mutant deficient in paralogous gene to *lguB*, *llg_31080*; and yellow, mutant deficient in paralogous gene to *lguE*, *llg_41580.* Values represent average ± S.D. of three independent cultures.

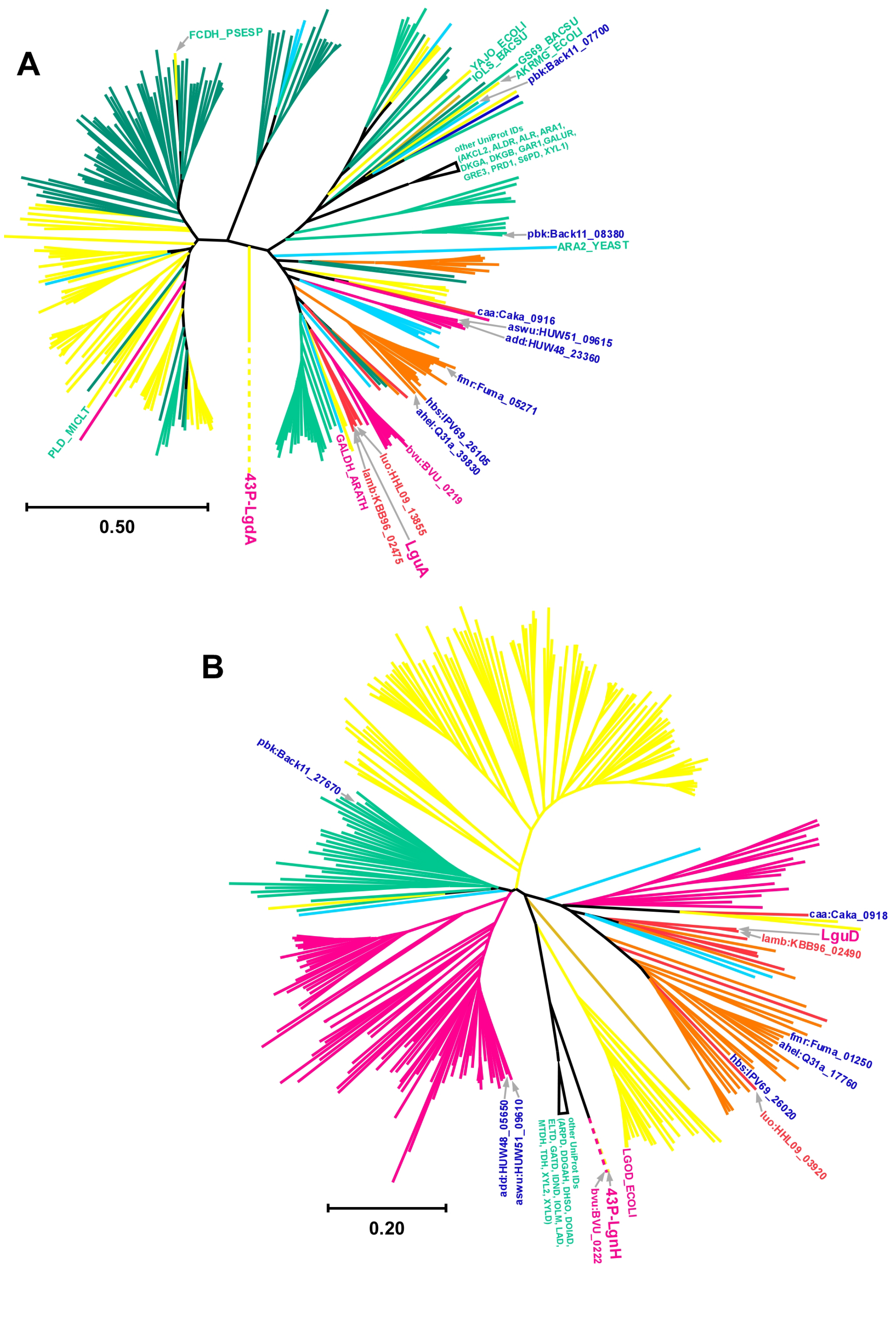
A
FCDH_PSESP
YAJO_ECOLI
IOLS_BACSU
GS69_BACSU
AKRMG_ECOLI
pbk:Back11_07700
other UniProt IDs (AKCL2, ALDR, ALR, ARA1, DKGA, DKGB, GAR1,GALUR, GRE3, PRD1, S6PD, XYL1)
pbk:Back11_08380
ARA2_YEAST
caa:Caka_0916
aswu:HUW51_09615
add:HUW48_23360
fmr:Fuma_05271
hbs:IPV69_26105
ahel:Q31a_39830
bvu:BVU_0219
luo:HHL09_13855
LguA
lamb:KBB96_02475
GALDH_ARATH
43P-LgdA
PLD_MICLT
0.50
B
pbk:Back11_27670
caa:Caka_0918
LguD
lamb:KBB96_02490
fmr:Fuma_01250
ahel:Q31a_17760
hbs:IPV69_26020
luo:HHL09_03920
LGOD_ECOLI
43P-LgnH
bvu:BVU_0222
other UniProt IDs (ARPD, DDGAH, DHSO, DOIAD, ELTD, GATD, IDND, IOLM, LAD, MTDH, TDH, XYL2, XYLD)
aswu:HUW51_09610
add:HUW48_05650
0.20

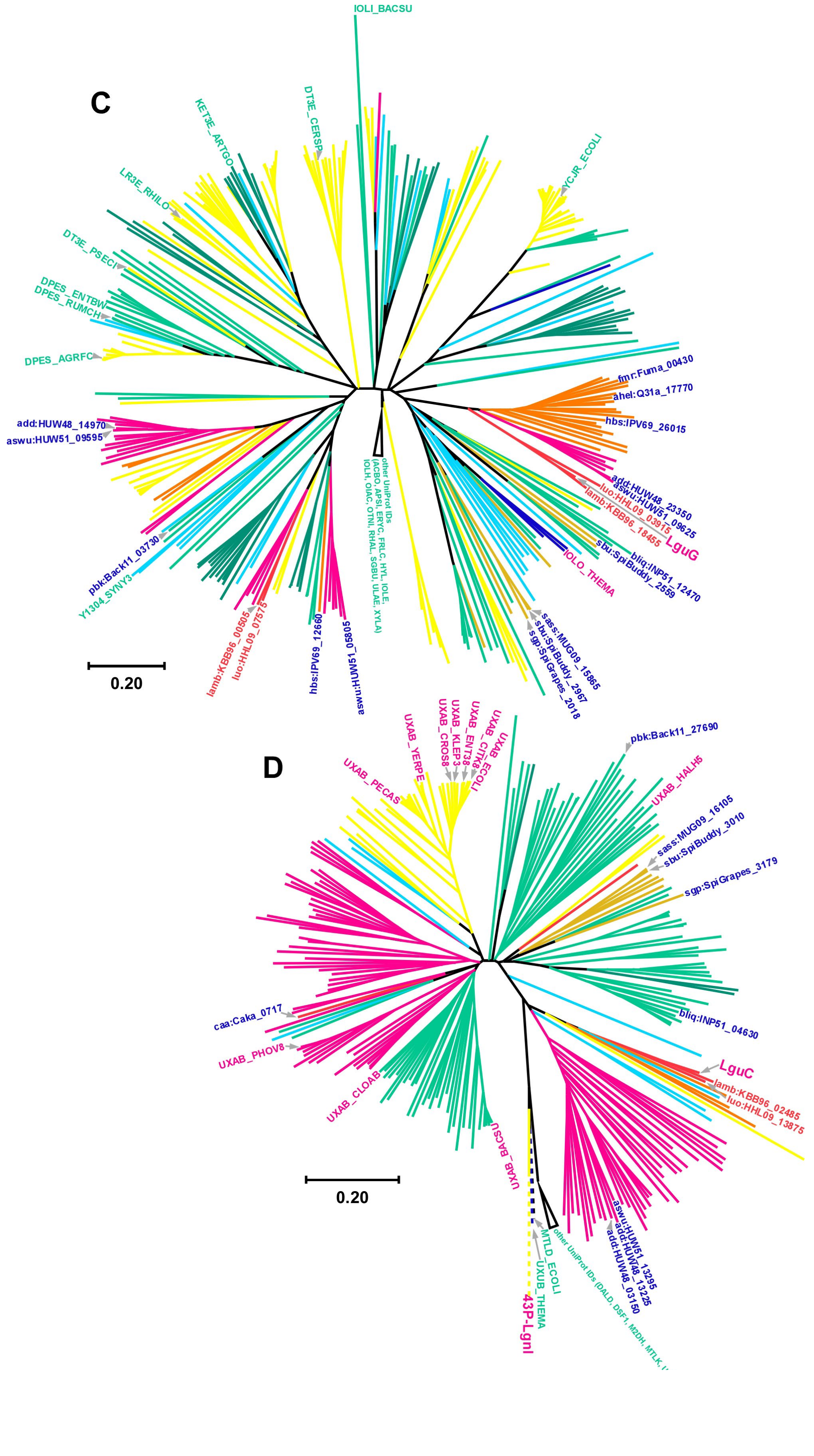
C
IOLI_BACSU
KET3E_ARTGO
DT3E_CERSP
LR3E_RHILO
YCJR_ECOLI
DT3E_PSECI
DPES_ENTBW
DPES_RUMCH
DPES_AGRFC
fmr:Fuma_00430
ahel:Q31a_17770
hbs:IPV69_26015
add:HUW48_14970
aswu:HUW51_09595
add:HUW48_23350
aswu:HUW51_09625
luo:HHL09_03915
lamb:KBB96_18455
LguG
sbu:SpiBuddy_2559
bliq:INP51_12470
IOLO_THEMA
pbk:Back11_03730
Y1304_SYNY3
other UniProt IDs (ACBO, APSI, ERYC, FRLC, HYI, IOLE, IOLH, OIAC, OTNI, RHAL, SGBU, ULAE, XYLA)
sass:MUG09_15865
sbu:SpiBuddy_2967
sgp:SpiGrapes_2018
lamb:KBB96_00505
luo:HHL09_07575
hbs:IPV69_12660
aswu:HUW51_05805
0.20
D
UXAB_PECAS
UXAB_YERPE
UXAB_CROS8
UXAB_KLEP3
UXAB_ENT38
UXAB_CITK8
UXAB_ECOLI
pbk:Back11_27690
UXAB_HALH5
sass:MUG09_16105
sbu:SpiBuddy_3010
sgp:SpiGrapes_3179
bliq:INP51_04630
caa:Caka_0717
UXAB_PHOV8
UXAB_CLOAB
LguC
lamb:KBB96_02485
luo:HHL09_13875
UXAB_BACSU
aswu:HUW51_13295
add:HUW48_13225
add:HUW48_03150
MTLD_ECOLI
UXUB_THEMA
43P-LgnI
other UniProt IDs (DALD, DSF, M2DH, MTLK,
0.20

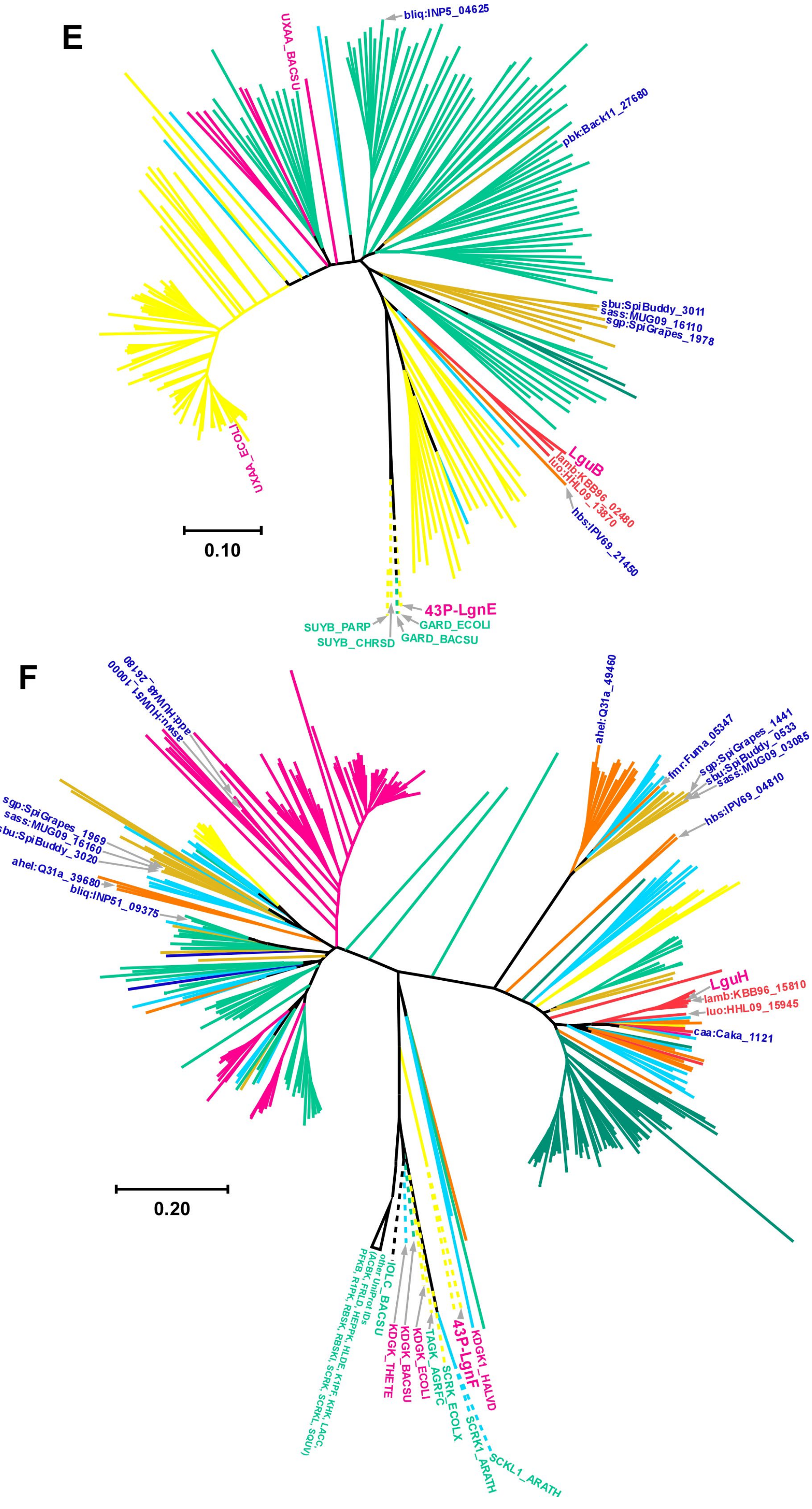

E
bliq:INP5_04625
UXAA_BACSU
pbk:Back11_27680
sbu:SpiBuddy_3011
sass:MUG09_16110
sgp:SpiGrapes_1978
UXAA_ECOLI
LguB
lamb:KBB96_02480
luo:HHL09_13870
hbs:IPV69_21450
0.10
43P-LgnE
SUYB_PARP
SUYB_CHRSD
GARD_ECOLI
GARD_BACSU
F
add:HUW48_26180
aswu:HUW51_10000
ahel:Q31a_49460
fmr:Fuma_05347
sgp:SpiGrapes_1441
sbu:SpiBuddy_0533
sass:MUG09_03085
hbs:IPV69_04810
sgp:SpiGrapes_1969
sass:MUG09_16160
sbu:SpiBuddy_3020
ahel:Q31a_39680
bliq:INP51_09375
LguH
lamb:KBB96_15810
luo:HHL09_15945
caa:Caka_1121
0.20
other UniProt IDs
(ACBK, FRLD, HEPPK, HLDE, K1PF, KHK, LACC,
PFKB, R1PK, RBSK, RBSKI, SCRK, SCRKL, SQUV)
IOLC_BACSU
KDGK_THETE
KDGK_BACSU
KDGK_ECOLI
TAGK_AGRFC
SCRK_ECOLX
43P-LgnF
SCRK1_ARATH
KDGK1_HALVD
SCKL1_ARATH

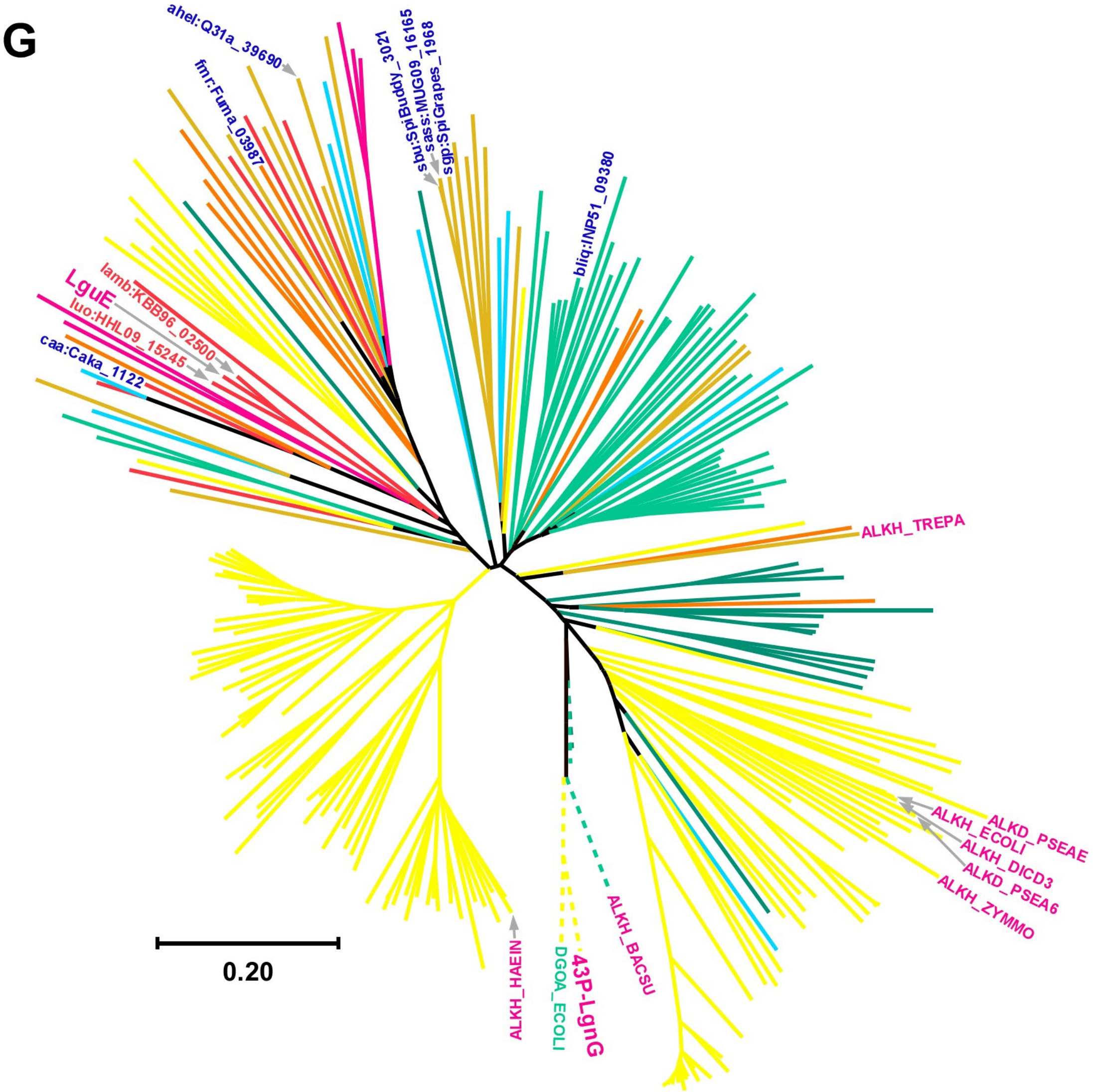


**Fig. S5. Phylogenetic trees based on LguA, LguD, LguG, LguC, LguB, LguH, and LguE amino acid sequences and related sequences from the KEGG genome and UniProt databases.** Panels A-G show phylogenetic trees for LguA **(A)**, LguD **(B),** LguG **(C)**, LguC **(D)**, LguB **(E)**, LguH **(F)**, and LguE **(G).** UniProt database sequences are shown by green or magenta letters with accession numbers. Organisms having all seven or five orthologs from the KEGG database are shown in red or blue letters, with KEGG organisms and accession numbers. Enzymes having the same functions as Lgu proteins and corresponding enzymes from strain 43P are shown in magenta, and those from the UniProt database that have other functions are shown in green. Line colors denote affiliations of organisms at the phylum level; red, *Verrucomicrobiota*; orange, *Planctomycetota*; magenta, *Bacteroidota*; dark yellow, *Spirochaetota*; green, *Bacillota*; dark green, *Actinomycetota*; yellow, *Pseudomonadota*; blue, *Thermotogota*; and cyan, other phyla including *Archaea* and *Eukaryota*. In (**A-D**) and (**F**), branches containing sequences from the UniProt database that had other functions only are manually compressed and indicated as “other UniProt IDs” with enzyme functions (prefix of IDs) in parentheses. Dashed lines indicate that the lengths are shortened by 1/4, and therefore do not reflect the actual phylogenetic distances.

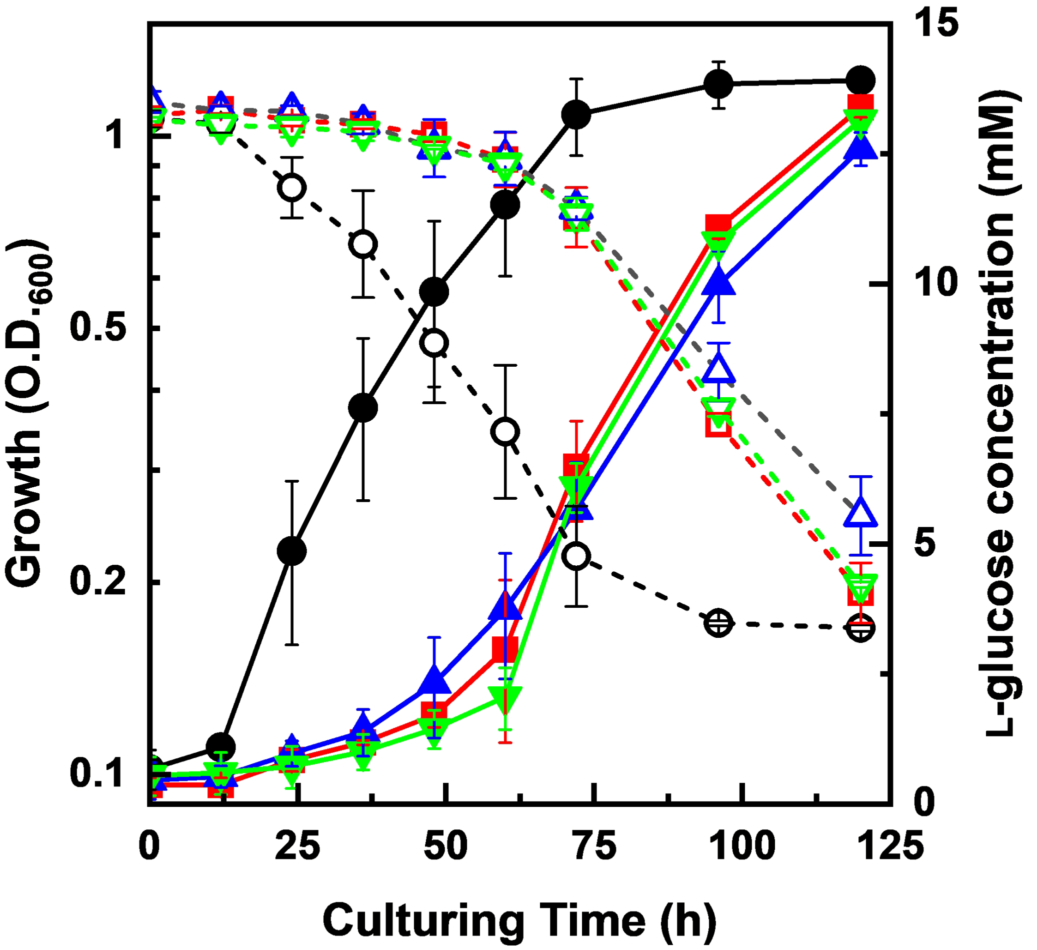


**Fig. S6. Growth and L-glucose consumption of LG18, *L. luteus*, *L. ambystomatis* and *L. arcticus* in L-GlcMM.** Growth and L-glucose consumption are indicated by closed and open symbols, respectively. Results for LG18, *L. luteus*, *L. ambystomatis* and *L. arcticus* are indicated by black circles, green triangles, red squares and blue triangles, respectively. Values represent average ± S.D. of three independent cultures.
